# A genomic tale of inbreeding in western Mediterranean human populations

**DOI:** 10.1007/s00439-025-02747-9

**Published:** 2025-05-10

**Authors:** Candela L. Hernández, Luis J. Sánchez-Martínez, Francisco C. Ceballos, Jean M. Dugoujon, Luisa Pereira, Rosario Calderón

**Affiliations:** 1 Departamento de Biodiversidad, Ecología y Evolución, Facultad de Ciencias Biológicas, Universidad Complutense de Madrid, Madrid, Spain; 2 Instituto de Salud Carlos III, Santiago de Compostela, 15706 Spain; 3 CNRS UMR 5288 Laboratoire d’Anthropologie Moléculaire et d’Imagerie de Synthèse (AMIS), Université Paul Sabatier Toulouse III, Toulouse, France; 4 i3S, Instituto de Investigação e Inovaçãao em Saúde, Universidade do Porto, Porto, Portugal; 5 Ipatimup, Instituto de Patologia e Imunologia Molecular da Universidade do Porto, Porto, Portugal

## Abstract

Consanguineous marriages are common in many worldwide human populations, and the biological consequences for offspring can be relevant at the biomedical level. The current genomic revolution displayed through genome-wide studies is challenging the paradigm in the analysis of consanguinity. Here, we analyzed genomic inbreeding patterns in human populations located at the western edge of the Mediterranean region (Iberia and Morocco). Runs of Homozygosity (ROH) (*autozygosity* fragments) were identified in 139 autochthonous individuals originating from southern Iberia and Morocco via microarray data. All individuals analyzed carried at least one ROH in their genomes. The genomic inbreeding coefficient (*F*_ROH_) and the presence of ROH islands (ROHi) revealed interesting patterns in the target populations as well as in the rest of the Mediterranean basin. Moroccan Berbers presented signals of recent inbreeding, relying on high coverage of long ROH (> 5 Mb) and *F*_ROH_. The location and structure of ROHi among people in the western Mediterranean could be interpreted as a signature of common genetic links across the Strait of Gibraltar. We found a significant enrichment of some relevant biological functions in the estimated ROHi hotspots associated with the immune system and chemosensation. Genomic inbreeding approaches allow us to understand past population histories and can be used as a proxy to scan the genome in search of selection signals.

**Supplementary Information:**

The online version contains supplementary material available at 10.1007/s00439-025-02747-9.

## Introduction

The consanguineous process and its impact in a population genetic and biomedical context have been extensively studied in many human populations at different geographical scales (Cavalli-Sforza and Bodmer 1971; Moroni et al. 1972; Pettener 1985; Calderón 1989; Calderón et al. 1993; Bittles and Neel 1994; Bittles et al. 2001; Modell and Darr 2002; Alfonso-Sánchez et al. 2004; Cavalli-Sforza et al. 2004; Calderón et al. 2005; Tadmouri et al. 2009; Hamamy et al. 2011; Romeo and Bittles 2014; Romdhane et al. 2019; among others). One of the main effects of recurrent consanguineous behavior across generations consists of the variation in the expected genotypic frequencies under Hardy‒Weinberg equilibrium in the population. Thus, the frequency of homozygous genotypes increases because parents of inbred offspring may share the same copy of an allele identical by descent (IBD) inherited from a common ancestor. Therefore, the probability of occurrence of any pair of IBD alleles in an inbred individual is called the inbreeding coefficient, *F*, and the genotype is *autozygous* (Hedrick 2005; Hartl and Clark 2006).

Many population surveys have revealed that the incidence of consanguinity is very heterogeneous within and between continents. Currently, the frequency of consanguineous marriages in western Europe is very low (~ 1% of total marriages in the population), whereas in communities from North Africa, the Near East/Middle East, western Asia and India, with deep rooting cultural rules, traditions and religions, this type of mating, mainly between first cousins, continue to be socially encouraged (30–50% of total marriages in the general population) (Hamamy et al. 2011).

The biomedical consequences of inbreeding can be significant. In human populations with relatively high and long-standing incidence rates of consanguineous unions, offspring can be affected by a higher incidence of recessive Mendelian disorders, multiple birth defects, and a significant pre-reproductive mortality rate, among other outcomes (Bittles and Neel 1994; Christianson et al. 2006; Alvarez et al. 2009; Bittles and Black 2010; Giacopuzzi et al. 2017).

Estimates of the inbreeding coefficient, *F*, have traditionally been based on pedigrees via Wright’s path method (Wright 1922). Later, other sources of information, such as population surnames, were used as proxies for biological relatedness (Weiss 1980; Lasker 1985, 1988). Nevertheless, the advent of genome-wide scanning technologies, such as single-nucleotide polymorphism (SNP) microarrays and high-throughput sequencing, has opened new perspectives in the study of consanguinity. Thus, new approaches to parental relatedness no longer rely only on historical records but rather on biological data itself. This is accomplished through the identification of homozygous genomic segments, termed *Runs of Homozygosity* (ROH). ROH occur when inbred individuals inherit from both parents two identical genomic segments from a common ancestor (McQuillan et al. 2008). Thus, ROH are IBD chromosomal segments distributed across the genome by tracts of DNA.

To quantify genomic inbreeding coefficients, two main approaches are used: single-point and multi-point procedures. The former is based on the excess homozygosity across each SNP; the latter, however, accounts for the genome as a whole rather than each SNP individually. This approach is the most commonly used method to detect inbreeding from human genomic data (Keller et al. 2011; Gazal et al. 2014). Accordingly, it can be inferred that the greater the degree of biological relatedness between spouses is, the greater the number of ROH fragments generated in the offspring genome. ROH fragments are broken by recombination across generations, giving rise to a greater number of fragments with smaller sizes (Gibson et al. 2006). Thus, recombination processes allow one to distinguish between recent and more ancient consanguinity based on autozygous tract lengths (Kirin et al. 2010; Nothnagel et al. 2010; Pemberton et al. 2012; Ceballos et al. 2018; Ferreira et al. 2022; among others).

The first human population studies focused on identifying and characterizing ROH to assess the magnitude of genomic inbreeding revealed that ROH fragments are common and longer than expected in outbred populations (Gibson et al. 2006; Li et al. 2006; McQuillan et al. 2008). Isolated groups with a more than predictable consanguinity therein retain lower levels of allelic heterogeneity due to a more relatively homogeneous genotypic background. This phenomenon affects the *F*_ROH_, with a significant increase in the number of large ROH segments. These scenarios correlate with population consanguinity levels (Karafet et al. 2015; Kang et al. 2016; Ceballos et al. 2019).

Currently, it is assumed that modern genome-wide scan technologies have the potential to achieve a more reliable, accurate assessment of the real extent of homozygosity in the analyzed genomes by characterizing several hundred thousand or few millions of SNPs. Nevertheless, the absence of standardization of ROH calling parameters and the density differences across microarrays represent the main caveats in exploring the history of human populations. This fact renders direct comparison between studies unreliable. Thus, PLINK input settings together with linkage disequilibrium and minor allele frequency pruning can notably impact ROH results for medium-density genotypes (Meyermans et al. 2020). In addition, the use of a complex variety of SNP chips, which analyze different variants in a variable number, and the non-uniformity in bioinformatics criteria to define ROH with specific ROH lengths across the genome clearly affect ROH identification. For example, when a panel of 3 million SNPs is used, which enables the reliable detection of ROH as short as 100 kb, the mean total ROH length among Han Chinese individuals is 510 Mb, whereas the cumulative length of 130 Mb is inferred via the use of 400,000 SNPs (Frazer et al. 2007). Finally, a certain heterogeneity with respect to how an ROH fragment is denoted, such as a genetic distance (centimorgans, cM) or physical distance, also exists (Auton et al. 2009; Skourtanioti et al. 2023). In spite of that, Kirin et al. (2010), studying several globally representative native populations from different continental areas, provided interesting results concerning the impact that distinctive evolutionary and demographic population histories would have had on the architecture of their own ROH profiles and, consequently, on the extent of the inbreeding coefficient, *F*_ROH_. Interestingly, when ROH > 1.5 Mb were analyzed, *F*_ROH_ correlated most strongly (*r* = 0.86) with the inbreeding coefficient obtained from an accurate seven-generation pedigree. Using extended pedigrees of royal European dynasties with complex inbreeding loops, it has been found that above the 10th generation, the change in the inbreeding coefficient is less than 1% (Alvarez et al. 2009).

Moreover, population studies have shown clear differences in ROH features such as the number and total length of ROH (Kirin et al. 2010; Nothnagel et al. 2010; Pemberton et al. 2012). These findings suggest that specific ROH structures observed in a gene pool are the consequence of demographic events in the genome across generations (Ceballos et al. 2018). Therefore, the genomic phenomenon of ROH is related to consanguinity, population size and endogamy, representing an authentic way to infer past population behaviors.

Currently, the population load caused by multifactorial diseases attributed to consanguinity is the subject of lively debate (Romdhane et al. 2019; Malawsky et al. 2023). For example, in case‒control studies of patients with schizophrenia, a group of ROH fragments was significantly more common than in the genomes of cases (Lencz et al. 2007; Keller et al. 2012). Other complex diseases are associated with ROH burden (e.g., Alzheimer’s disease, Parkinson’s disease, Hodgkin’s lymphoma and other types of cancer) (Bacolod et al. 2008; Simón-Sánchez et al. 2012; Wang et al. 2013; Ghani et al. 2015; Thomsen et al. 2016; Moreno-Grau et al. 2021). These studies suggest that the largest number of ROH fragments represents a major contributor to complex disease risk, although this supposition is still debatable. This particularity seems to be responsible for deleterious homozygosity scenarios.

Another interesting and modern approach involves the analysis of ROH distribution across the human genome, which appears to be nonrandom (Curtis et al. 2008). Similarly, there is evidence of genome regions where ROH are more common than expected (ROH *islands*, ROHi or *hotspots*) (Pemberton et al. 2012; Ceballos et al. 2018). The level of homozygosity in ROHi would be greater around a certain *locus* than around the rest of the genome. The uneven concentration of autozygous fragments across the genome may be due to either the randomness of recombination events or demographic event effects on human genetic diversity. However, ROHi could also be a potential signaling target of positive selection. This means that a reduction in the level of genetic diversity, as a consequence of the increase in homozygosity around selected *loci* would indeed represent adaptive responses (Nothnagel et al. 2010; Pemberton et al. 2012). ROHi investigations are extremely common in animal genomics for identifying *loci* linked to economically relevant traits in livestock (Mastrangelo et al. 2018; Peripolli et al. 2018; Grilz-Seger et al. 2019; Gorssen et al. 2021; Stoffel et al. 2021). However, in human populations, data are still scarce (Ceballos et al. 2019).

In recent years, important efforts have been made to analyze ROH patterns in specific human populations, mainly from North Africa and southwestern Asia (Ferreira et al. 2022), given the persistent and still relevant consanguineous behavior across generations (Elliott et al. 2022; Mezzavilla et al. 2022). Interestingly, however, knowledge of the genomic inbreeding patterns of European populations is still limited.

Here, we report the first detailed analysis of western Mediterranean (WM) populations –from southern Iberia and northern Morocco– to investigate: *i*) the genomic architecture of ROH and its distribution throughout the genome, *ii*) the genomic inbreeding levels, *F*_ROH_, and *iii*) the presence, chromosome location and extent of ROHi. The potential genetic differentiation within and between Iberian and Moroccan Berbers subpopulations is assessed and interpreted in terms of demographic history, population structure and cultural patterns. The present population genetic work is integrated with other Mediterranean populations embracing other regions of this geographic space. The WM area, with the Strait of Gibraltar (the closest physical connection between Europe and Africa) and its surroundings, has played a crucial role in early human migrations across continents from prehistoric times.

## Materials and methods

### Sampling collection and populations

Biological samples from total blood or cheek swabs were collected from healthy, unrelated and autochthonous subjects (at least three generations back) at the extreme edge of the WM, including the regions of Andalusia (southern Spain), southern Portugal and Morocco (northwest Africa). The sampling process was designed and carried out by the authors (RC, LP, JMD). More details can be found elsewhere (Pereira et al. 2005; Calderón et al. 2006; Coudray et al. 2009; Ambrosio et al. 2010). Each donor provided written informed consent, and protocols were approved by the Clinical Research Ethics Committee from San Carlos Clinical Hospital (ethics approval number 14/415-E_BS) and in accordance with the principles of the Declaration of Helsinki.

### Genomic analyses

The samples were genotyped with Illumina’s Omni 2.5–8 BeadChip array (~ 2.4 million SNP markers) as described previously by Hernández et al. (2020). The global analyzed sample consisted of 139 individuals: 35 from eastern Andalusia (Granada province), 35 from western Andalusia (Huelva province), 35 from southern Portugal, and 34 from Morocco (Berbers from Asni, *n* = 15; Bouhria, *n* = 10; Figuig, *n* = 9). The possible recent genetic relatedness among individuals was considered via the PC-Relate pipeline (Conomos et al. 2016).

For comparative purposes, we compiled genomic information from Iberia (IBS) (Spanish population), Italy (TSI) (1000 Genomes Project, 1KGP, phase 3, Auton et al. 2015) and five other populations settled across the Mediterranean space (HGDP last release, Bergström et al. 2020). In total, 519 individuals from 13 populations were included (Table S1). The quality control (QC) criteria, undertaken by using PLINK v.1.9 (Chang et al. 2015), considered removing samples or variants with missing genotype rate per person and per SNP, respectively (both > 5%), and variants with significant departure (*p*-value threshold: 0.001) from Hardy‒Weinberg equilibrium; identity by descent (IBD) was considered by generating *pihat* pairwise values between individuals in each population (all possible combinations yielded values < 0.5). The global number of variants identified after the merging and QC processes was ~ 1.8 million SNPs. The hg19/GRCh37 human reference sequence was used in all analyses.

### Identification of ROH and Estimation of the inbreeding coefficient (F_ROH_)

We used PLINK v1.9 to identify and characterize ROH in the genotype data of the six WM population samples. PLINK has been extensively used to call ROH in human genomic studies since its direct observational approach allows researchers to have full control of the ROH calling process. Using high-density panels which harbour different thousands of SNPs covering the whole genome, four main approaches have been proposed to estimate inbreeding coefficients which are based on: *i*) excess of homozygosity (*F*_HOM_) (Slate et al. 2004); *ii*) identity by state (IBS) using genomic relationship matrices (GRM) (*F*_GRM_) (VanRaden et al. 2011), *iii*) correlation of uniting gametes following Wright (1922), and *iv*) ROH (*F*_ROH_) (McQuillan et al. 2008; Ceballos et al. 2018). The latter seems to estimate more precisely the level of autozygosity of an individual’s genome or population that the inbreeding coefficient estimated from pedigrees (*F*_PED_) or other estimators. ROH parameter is believed to capture better inbreeding as it strongly correlate with homozygous correlation load (Keller et al. 2012), and also to identify genomic regions that are possibly under historical selection pressure (Kirin et al. 2010) (for a review, see Peripolli et al. 2017).

The ROH were identified on autosomes with the following arguments: -*homozyg-snp 30* (minimum number of SNPs that a ROH is required to contain); -*homozyg-kb 300* (length in Kb of the sliding window); -*homozyg-density 30* (required minimum density to consider a ROH); -*homozyg-gap 1000* (length in Kb between two SNPs to be considered in two different segments); -*homozyg-window-snp 30* (number of SNPs that the sliding window must contain); -*homozyg-window-het 1* (number of heterozygous SNPs allowed in a window); -*homozyg-window-missing 5* (number of missing calls allowed in a window) and -*homozyg-window-threshold 0.05* (proportion of overlapping windows that must be called homozygous to define a given SNP as being within a “homozygous” segment).

To analyze the ROH structure, we computed different parameters, taking into account both the total number of ROH (NROH) and the total sum of the ROH (SROH) length separated into six length categories: ROH1 (ROH 0.3–0.5 Mb), ROH2 (ROH 0.5-1 Mb), ROH3 (ROH 1–2 Mb), ROH4 (ROH 2–4 Mb), ROH5 (ROH 4–8 Mb), and ROH6 (ROH > 8 Mb).

*F*_ROH_ measures the actual proportion of the autosomal genome that is autozygous over and above a specific minimum length ROH threshold. *F*_ROH_, which uses a genomic approach, captures the total inbreeding coefficient (*F*_IT_) within the resolution of the data available and the size of the ROH that can be called (McQuillan et al. 2008).

$${F_{{\text{ROH}}}} = \frac{{\mathop \sum \nolimits_{i = 1}^n ROH > 1.5Mb}}{{3Gb}}$$

Wright’s inbreeding coefficient (*F*_IS_), which measures the reduction in heterozygosis (H) of an individual due to nonrandom mating within a subpopulation, was obtained via the *--het* flag in PLINK:

$$\:{F}_{\text{I}\text{S}}=\frac{O\left(HOM\right)-E\left(HOM\right)}{N-E\left(HOM\right)}$$

where *O* (*observed hom*) is the observed number of homozygous SNPs, *E* (*expected hom*) is the expected number of homozygous SNPs considering H–W proportions, and N is the total number of nonmissing genotyped SNPs.

The distribution of ROH per chromosome was assessed considering two levels: ROH fragments > 0.30 Mb and > 1.5 Mb for the global WM sample and independently for the Iberian and Moroccan population samples. Data from the Genome Reference Consortium were used to estimate the size of human chromosomes (https://www.ncbi.nlm.nih.gov/grc/human/data).

To illustrate the extent of relationships between the total ROH length, ROH number by category and *F*_ROH_ length values per individual, a Spearman correlation matrix was computed with hierarchical clustering. A linear mixed model (LMM) of random effects was then used to calculate the weight of each ROH length type when assessing the level of genomic inbreeding (*F*_ROH_) in WM populations. In the statistical model used, populations were treated as random effects by using the *nlme* R package (Pinheiro et al. 2023). The resulting standardized coefficient measures the relative weight of each category on the variable of interest (*F*_ROH_).

### Studying the origin of autozygosity

In this work, we followed two different but complementary approaches to determine the ROH’s origin: *i*) Number of ROH vs. the sum of ROH length plot: following McQuillan et al. (2008), if an individual has a higher excess of SROH > 1.5 Mb than NROH > 1.5 Mb, we detect autozygosity by consanguinity. However, if both SROH > 1.5 Mb and NROH > 1.5 Mb are high, it suggests autozygosity driven by genetic drift, and *ii*) *F*_ROH_*vs. F*_IS_ plot: the position of populations in this plot is analyzed with respect to the diagonal line (*F*_IS_ = *F*_ROH_) and the horizontal line (*F*_IS_= 0). Thus, (a) populations (or individuals) close to the diagonal line exhibit a strong tendency toward systematic inbreeding or *F*_IS_, primarily due to deviation from panmixia; (b) populations situated close to the *F*_IS_= 0 line are more affected by panmictic inbreeding resulting from genetic drift; and (c) when *F*_IS_ is negative, low Ne, isolation, and genetic drift become crucial factors.

### ROH Islands and genomic distribution

ROH islands (ROHi) are defined as regions in the genome where the proportion of individuals of a population is greater than expected by a binomial distribution. To search for ROHi, a sliding window of 100 kb was used. In every 100-kb genomic window, the number of people with ROH was obtained. To determine whether a specific genomic window has a significant enrichment of ROH across the population, a binomial test with a *p-*value < 2 × 10^− 5^ with Bonferroni correction for 2500 windows was applied. To compare ROHi between groups, it was considered that two ROHi from two different populations are indeed the same ROH if they share at least 75% of their length. Protein-coding genes present in ROHi were obtained using the *biomaRt* R package (Durinck et al. 2009) and the Ensembl database (hg19 genome version). *Gene Ontology* (GO) enrichment analysis was subsequently performed. Lists of genes contained in each island were inspected to determine which GO terms were overrepresented, if any (https://geneontology.org/). The results were retrieved with a false discovery rate (FDR) *p-*value < 0.05 and are shown only for those registering the top-fold enrichment values (≥ 10). Gene set enrichment analysis was applied to those ROHi especially frequent within the populations (present in at least 90% of the individuals) and those ROHi covering ≥ 1.5 Mb. We also inspected the occurrence of islands overlapping with those reported in previous studies (Nothnagel et al. 2010; Pemberton et al. 2012; Ceballos et al. 2019) to check for potential conserved regions of autozygosity.

We first applied this pipeline to each population as defined in Table S1 (*islands_step1*). To overcome the technical caveats given the differences between sample sizes, we then grouped the populations by defining three metapopulations from a geographical standpoint (*islands_step2*): western Mediterranean, *WM* (SPWA, SPEA, SPOR, MAAS, MABO, MAFI; total *n* = 139), central Mediterranean, *CM* (TSI, SARD; *n* = 120) and eastern Mediterranean, *EM* (ISBE, ISDR, ISPA; *n* = 134).

## Results

We identified a total of 81,592 ROH segments in the six studied WM population samples. The total and mean number of ROH per individual genome, the sum of the total ROH length (Mb) at both the individual and population levels, and the mean ROH length in each defined ROH length class per population are reported in Table 1. All individuals analyzed carried at least one ROH in their genomes. For comparison purposes, the same parameters estimated above are provided in Table S2 for the selected set of other additional Mediterranean populations.

**Table 1:** Summary statistics regarding ROH fragments in southern Iberian and Moroccan Berber populations. The total/mean ROH values of each variable per individual and population are shown in bold

Western Mediterranean Populations	*n*	ROH Length Category (Mb)	ROH Category	Number of individual (%)	Total ROH Number (%)	Mean Number of ROH / per individual	Total ROH Length (Mb)/population (%)	Total ROH Length (Mb)/per individual	Mean ROH Length (Mb)
**Iberian Peninsula** **Eastern Andalusians (Granada)**	35	0.3–0.5	ROH1	35/35 (100)	14,271 (65.59)	407.74	5411.68 (46.56)	154.62	0.38
		0.5-1	ROH2	35/35 (100)	6216 (28.57)	177.60	4091.61 (35.21)	116.90	0.66
		1–2	ROH3	35/35 (100)	1053 (4.84)	30.09	1360.82 (11.71)	38.88	1.29
		2–4	ROH4	35/35 (100)	170 (0.78)	4.86	435.08 (3.74)	12.43	2.56
		4–8	ROH5	28/35 (80.00)	39 (0.18)	1.11	211.20 (1.82)	6.03	5.41
		> 8	ROH6	7/35 (20.00)	8 (0.04)	0.23	111.71 (0.96)	3.19	13.96
Totals					**21,757**	**621.63**	**11622.10**	**332.06**	**0.53**
**Iberian Peninsula** **Western Andalusians (Huelva)**	35	0.3–0.5	ROH1	35/35 (100)	14,293 (65.25)	408.37	5412.04 (44.91)	154.63	0.38
		0.5-1	ROH2	35/35 (100)	6292 (28.72)	179.77	4145.74 (34.41)	118.45	0.66
		1–2	ROH3	35/35 (100)	1069 (4.88)	30.54	1374.31 (11.41)	39.27	1.29
		2–4	ROH4	35/35 (100)	171 (0.78)	4.89	452.66 (3.76)	12.93	2.65
		4–8	ROH5	30/35 (85.71)	56 (0.26)	1.60	315.76 (2.62)	9.02	5.64
		> 8	ROH6	14/35 (40.00)	24 (0.11)	0.96	347.86 (2.89)	9.94	14.49
Totals					**21,905**	**625.86**	**12048.36**	**344.24**	**0.55**
**Iberian Peninsula** **Southern Portuguese**	35	0.3–0.5	ROH1	35/35 (100)	14,031 (66.15)	400.89	5302.19 (47.05)	151.49	0.38
		0.5-1	ROH2	35/35 (100)	5979 (28.18)	170.83	3960.24 (35.14)	113.15	0.66
		1–2	ROH3	35/35 (100)	1004 (4.73)	28.69	1262.25 (11.20)	36.06	1.26
		2–4	ROH4	35/35 (100)	158 (0.74)	4.51	406.06 (3.60)	11.60	2.57
		4–8	ROH5	27/35 (77.14)	30 (0.14)	0.86	163.87 (1.45)	4.68	5.46
		> 8	ROH6	9/35 (25.71)	12 (0.06)	0.34	175.66 (1.56)	5.02	14.64
Totals					**21,214**	**606.11**	**11270.26**	**322.01**	**0.53**
**Asni Moroccan Berbers**	15	0.3–0.5	ROH1	15/15 (100)	4768 (63.76)	317.87	1806.22 (37.56)	120.46	0.38
		0.5-1	ROH2	15/15 (100)	2141 (28.63)	142.73	1418.38 (29.49)	94.56	0.66
		1–2	ROH3	15/15 (100)	408 (5.46)	27.20	524.88 (10.91)	34.99	1.29
		2–4	ROH4	15/15 (100)	94 (1.26)	6.27	250.42 (5.21)	16.69	2.66
		4–8	ROH5	13/15 (86.67)	30 (0.40)	2.00	162.60 (3.38)	10.84	5.42
		> 8	ROH6	9/15 (60)	37 (0.49)	2.47	645.69 (13.43)	43.05	17.45
Totals					**7478**	**498.53**	**4808.89**	**320.59**	**0.64**
**Bouhria Moroccan Berbers**	10	0.3–0.5	ROH1	10/10 (100)	3132 (65.33)	313.20	1186.21 (36.06)	118.62	0.38
		0.5-1	ROH2	10/10 (100)	1298 (27.08)	129.80	862.85 (26.23)	86.28	0.66
		1–2	ROH3	10/10 (100)	246 (5.13)	24.60	315.16 (9.58)	31.52	1.28
		2–4	ROH4	10/10 (100)	63 (1.31)	6.30	171.63 (5.22)	17.16	2.72
		4–8	ROH5	9/10 (90)	24 (0.50)	2.40	133.14 (4.05)	13.31	5.55
		> 8	ROH6	5/10 (50)	31 (0.65)	3.10	620.22 (18.86)	62.02	20.00
Totals					**4794**	**479.40**	**3289.22**	**328.92**	**0.69**
**Figuig Moroccan Berbers**	9	0.3–0.5	ROH1	9/9 (100)	2776 (62.46)	308.44	1051.46 (34.19)	116.83	0.38
		0.5-1	ROH2	9/9 (100)	1271 (28.60)	141.22	841.51 (27.36)	93.50	0.66
		1–2	ROH3	9/9 (100)	250 (5.63)	27.78	322.73 (10.49)	35.86	1.29
		2–4	ROH4	9/9 (100)	82 (1.85)	9.11	228.63 (7.43)	25.40	2.79
		4–8	ROH5	9/9 (100)	37 (0.83)	4.11	204.92 (6.66)	22.77	5.54
		> 8	ROH6	6/9 (66.66)	28 (0.63)	3.11	426.60 (13.87)	47.40	15.24
**Totals**					**4444**	**493.78**	**3075.84**	**341.76**	**0.69**

The abundance of short ROH is a characteristic of genomic organization in most contemporaneous human populations (McQuillan et al. 2008; Kirin et al. 2010; Nothnagel et al. 2010; Pemberton et al. 2012). This conclusion agrees with the percentage of short ROH segments (below 1 Mb) observed in the present survey, which was greater than 90% of the remaining ROH length types. This proportion is similar in both the Iberian and Berber groups. Similarly, the cumulative lengths of short ROH (including ROH1: 0.3–0.5 Mb and ROH2: 0.5-1 Mb) across the three Iberian samples revealed rather similar values, ranging from 151.49 to 154.63 Mb and 113.15-118.45 Mb, respectively. These results are again shared by Moroccan Berbers. Nevertheless, interesting dissimilarities are observed among southern Iberian samples when analyzing the presence of larger ROH segments (ROH5: 4–8 Mb, ROH6 > 8 Mb). This is the case for western Andalusians from Huelva, where differences in total ROH number and total ROH length were observed with respect to their relatives from eastern Andalusia (Granada) and southern Portugal (Table 1). In some manner, these ROH patterns would be similar between western Andalusians and Berbers. Moroccan Berbers are characterized by high coverage in longer ROH. The great majority of the Berber samples (20 out of 34: 58.82%) carried ROH fragments > 8 Mb (ROH6), with total ROH lengths across individual genomes varying from 43.05 Mb (Asni) to 62.02 Mb (Bouhria). Thus, the cumulative ROH length values represented ~ 3.2-fold more of their genomes in ROH6 (> 8 Mb) than those found in southern Iberians (i.e., 3.19 Mb, eastern Andalusians from Granada; 9.94 Mb, western Andalusians from Huelva). The reason for this apparent distinction between the Andalusian subpopulations may be that only a small portion of Granada subjects (8 out of 35) contained ROH6 (> 8 Mb) fragments in their genomes.

**Fig. 1:**
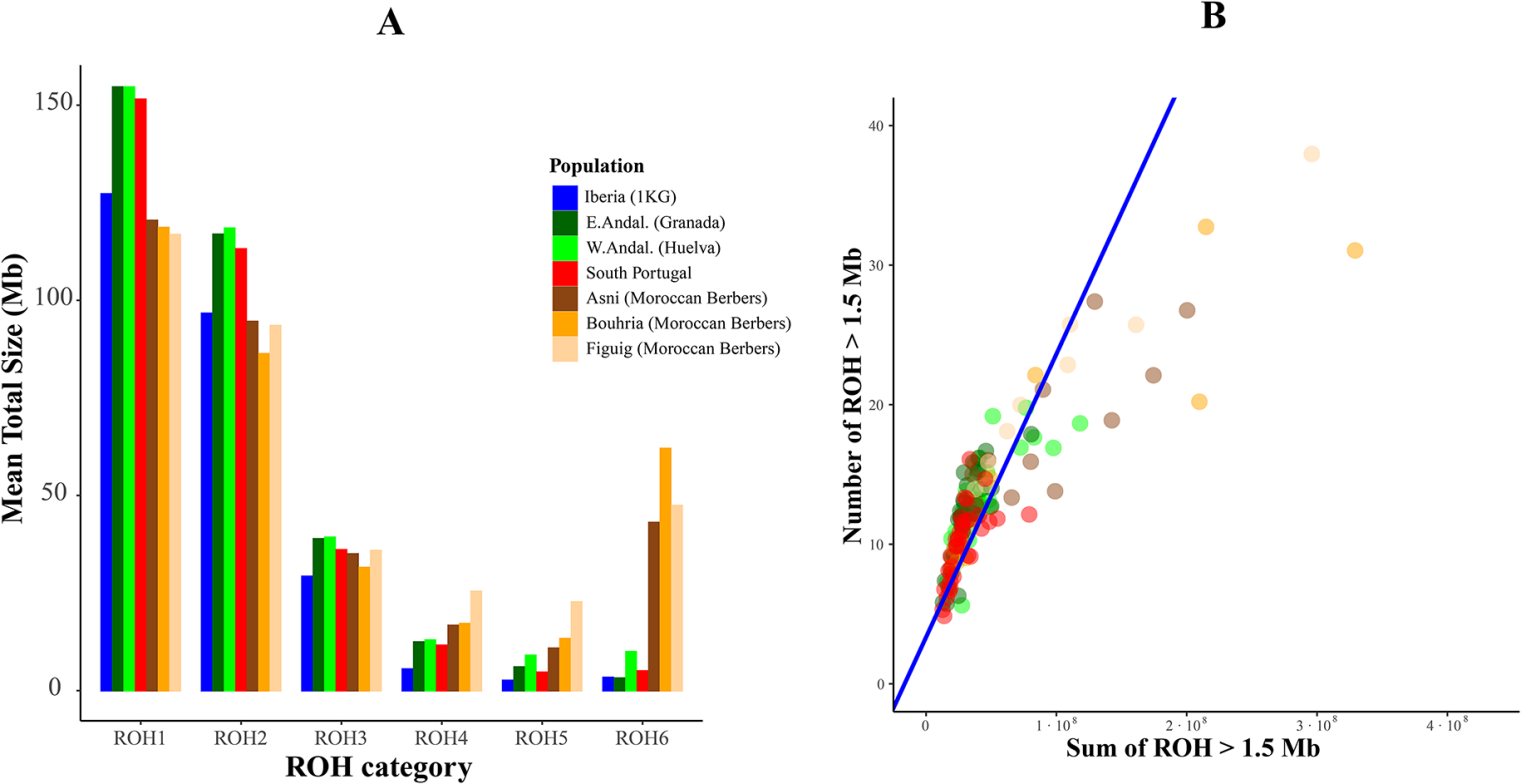
**A.** Mean ROH length (Mb) across the six stablished intervals of ROH sizes in western Mediterranean populations. Within each ROH length category, ROH were summed up and averaged per population. In the analysis, data from an Iberian (IBS) general sample (see Table S1) is plotted in indigo. **B** Relationship between sum of ROH length (Mb) (SROH) and number of ROH ≥ 1.5 Mb (NROH). Each point corresponds to an individual. ROH1 (ROH 0.3–0.5 Mb), ROH2 (ROH 0.5-1 Mb), ROH3 (ROH 1–2 Mb), ROH4 (ROH 2–4 Mb), ROH5 (ROH 4–8 Mb), and ROH6 (ROH > 8 Mb)

Figure 1A graphically shows the distribution patterns of the mean ROH lengths for each ROH category in all the examined WM subpopulations. 1): The extent of mean and standard deviation for each bin (i.e. population) is provided in Table S3. Genomic data from an Iberian (Spanish) general sample, IBS (*n* = 99), taken from the 1000 Genomes Project, have been included. Figure 1B depicts the relationships between the sum of the ROH length ≥ 1.5 Mb and the ROH number for each individual, where a positive and significant correlation (*r* = 0.8660, *p-*value > 0.001) is reached. The regression line that best fits the 1000 Genome Project IBS sample data is plotted in indigo. As shown, most of the Berber genomes exhibited greater spread across the upper right of the bidimensional space, standing apart from the Iberian subjects. The shown scenario is influenced by the presence of the longest ROH.

**Fig. 2:**
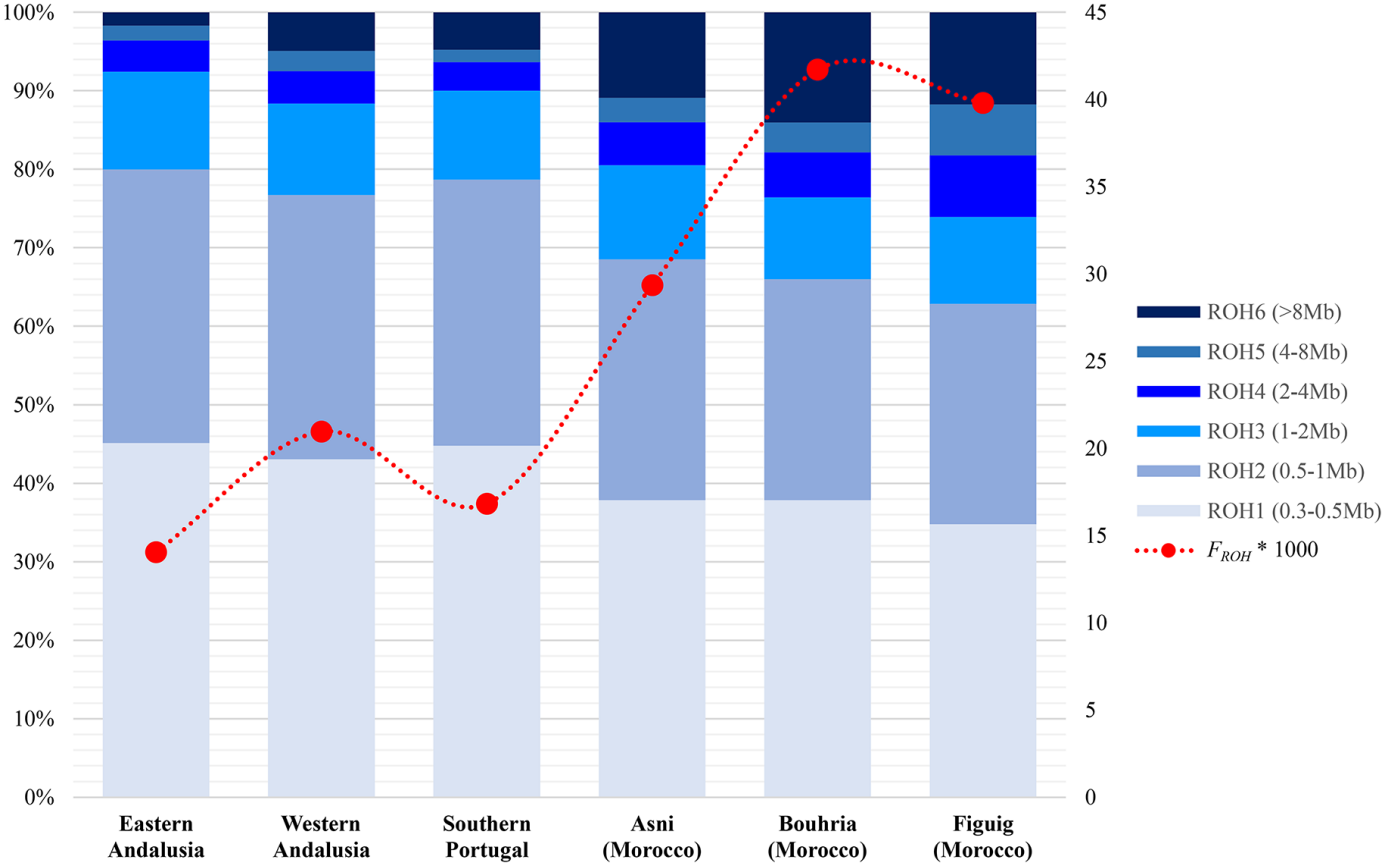
Proportions of total ROH length within each ROH length type in the analyzed WM populations. The estimated inbreeding coefficients (*F*_ROH_) are shown in red circles

The proportions of the total length of ROH across the six ROH classes together with the estimates of *F*_ROH_, through all ROH lengths ≥ 1.5 Mb, are graphically shown in Fig. 2. Consistent with what has been noted above, the estimated mean *F*_ROH_ value was almost twice as high in Berbers (*F*_ROH_ = 0.0369) than in Iberians (*F*_ROH_ = 0.0172). Specifically, Berbers from Bouhria and Figuig registered the greatest mean inbreeding levels, 0.0417 and 0.0398, respectively, as a reflection of the still highly prevalent consanguinity among North African populations. Among southern Iberians samples, western Andalusians registered the highest levels of *F*_ROH_= 0.0209.

**Fig. 3:**
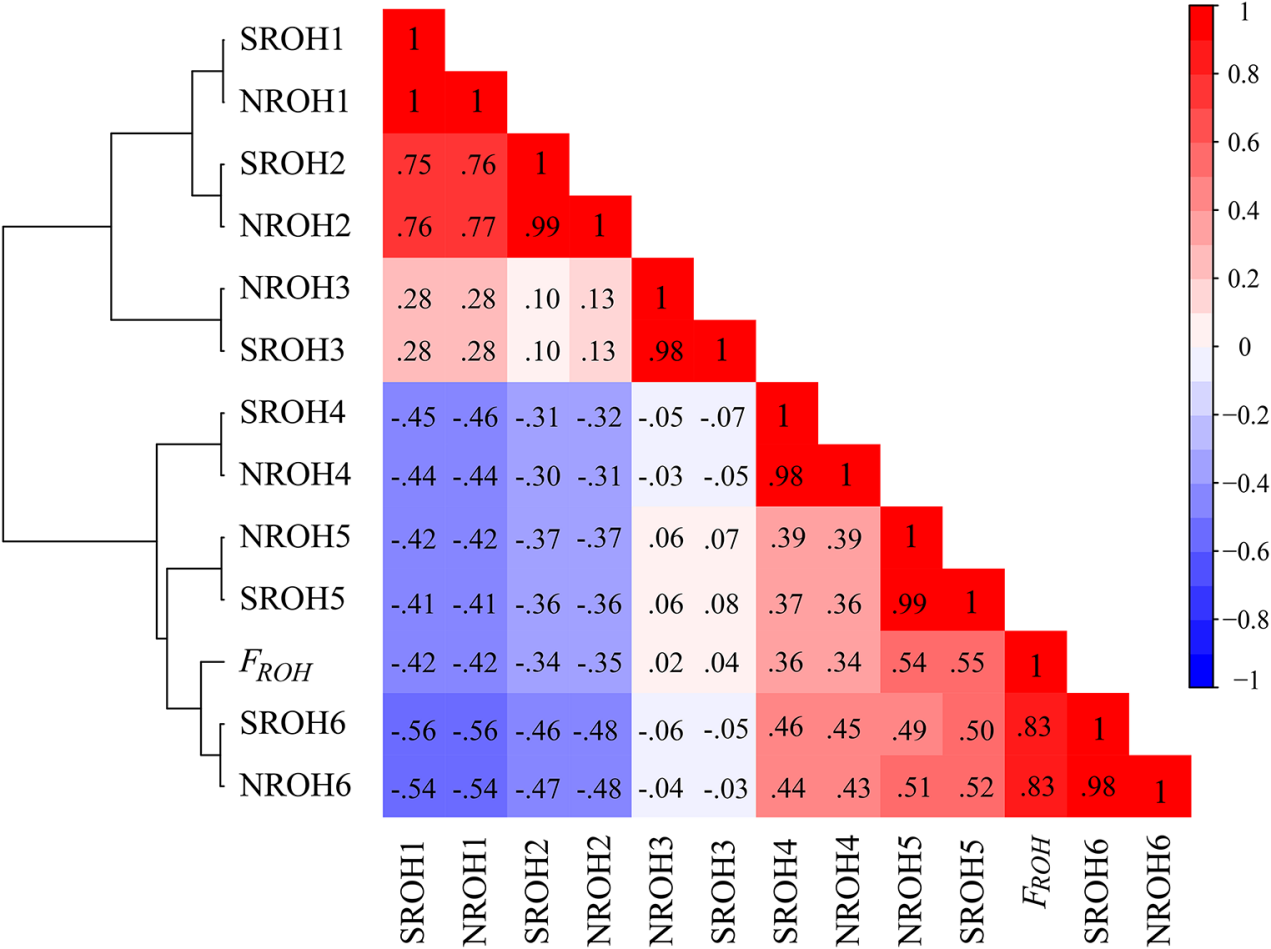
Heat-map of the pairwise coefficients of correlation (Spearman) based on NROH and SROH by categories and *F*_ROH_. The analyzed variables are hierarchically ordered on the left of the correlation matrix

In light of the observed results, attention should be given to the extent to which the genome architecture in ROH can influence the magnitude of *F*_ROH_. For this purpose, Spearman’s correlations between *F*_ROH_, NROH and SROH (in Mb) by ROH length categories were computed, and a heatmap was plotted based on the whole WM sample set (Fig. 3). The heatmap pattern is in accordance with the architecture of the generated dendrogram, showing two distinctive subclusters. One of them is defined by the ROH1 (0.3–0.5 Mb), ROH2 (0.5-1 Mb), and ROH3 (1–2 Mb) categories, and the other is defined by long ROH [ROH4 (2–4 Mb), ROH5 (4–8 Mb), and ROH6 (> 8 Mb)]. Strikingly, the ROH6 (> 8 Mb) class was most closely related to *F*_ROH_ (*r* = 0.83, *p-*value < 0.001). This interesting conclusion was similarly drawn when independently exploring the corresponding heatmaps from southern Iberians and Moroccan Berbers (data not shown here). A linear mixed model (LMM) of random effects was applied, and the results were fairly convergent. Specifically, a strong and significant effect of ROH length fragments on *F*_ROH_ among individuals was detected, particularly when ROH6 (> 8 Mb) segments were referenced (standardized coefficient value was 0.8056, *p*-value < 0.001).

**Fig. 4:**
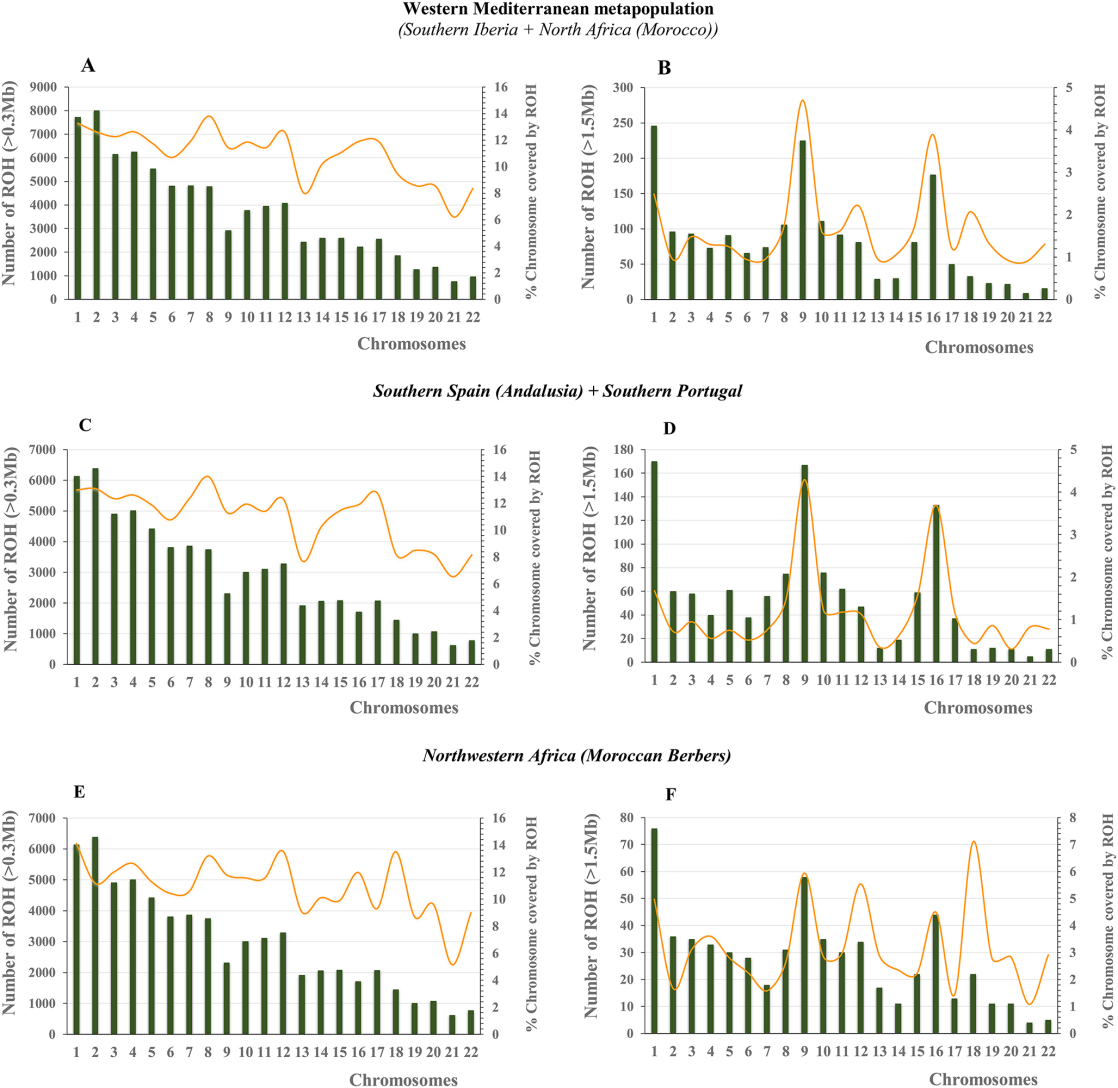
Distribution patterns of NROH ≥ 0.3 Mb (left column) and NROH ≥ 1.5 Mb (right column) across chromosomes. The yellow line shows the proportion of each chromosome covered by ROH. Plots are represented for the whole WM metapopulation as well as for southern Iberians and Moroccan Berbers

The distribution of ROH fragments within the human genome is not uniform, and its variation between chromosomes can be very wide (Pemberton et al. 2012). Figure 4 depicts the distribution patterns of ROH throughout the chromosomes (primary *x-*axis) against the mean proportion of each chromosome under ROH (secondary *y*-axis). Our interest was focused first on the WM sample and then on distinguishing southern Iberians from the group of Moroccan Berbers. The first block (left column) (ROH ≥ 0.3 Mb) shows, as expected, a sustained and decreasing tendency of the number of ROH with chromosome length. In contrast, the bar plot profiles in the second block (right column) seem to be highly influenced by the significant decrease in the amount of ROH ≥ 1.5 Mb in the human genome. Notably, the highest concentrations of ROH were observed on chromosomes 1 (C-1), C-9 and C-16 in both southern Iberian and Berbers. However, when the mean percentage coverage of ROH per chromosome is analyzed, the results are more dissimilar and heterogeneous. In general, North Africans presented higher mean proportions of chromosomes covered by ROH (total average: 3.18%) compared with Iberians (total average: 1.17%). The highest proportion of ROH coverage per chromosome (7.11%) was observed for C-18 in Berbers.

**Fig. 5:**
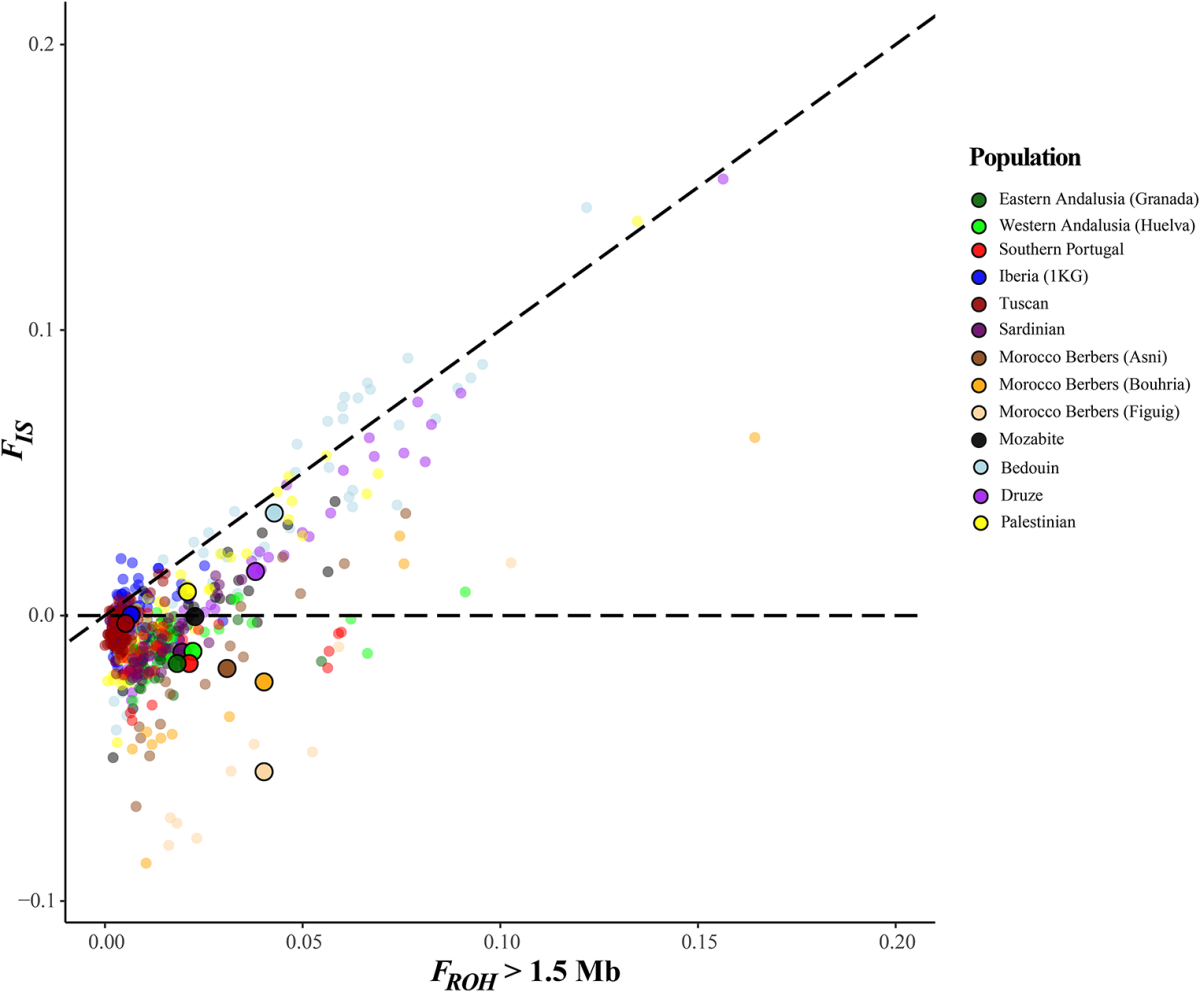
*F*_ROH_ ≥1.5 Mb and *F*_IS_ inbreeding coefficients are plotted for each individual from the database (see Table S1). Mean values are represented with solid, black-lined circles

The *F*_ROH_>1.5 Mb *vs. F*_IS_ inbreeding coefficients in Mediterranean populations are presented in Table S4 and graphically in Fig. 5. The plot shows how the majority of individuals are located close to and through the diagonal, with positive *F*_IS_ values correlated with high levels of *F*_ROH_. Nevertheless, the topology of the remaining subjects is mostly clustered near the bottom, close to the origin of the coordinates, as a consequence of the high incidence of short ROH. Thus, *F*_IS_ estimates could be structured into two categories: *i*) *F*_IS_ < 0, individuals apparently having excess of heterozygotes, and *ii*) 0 < *F*_IS_ < *F*_ROH_, individuals having consanguinity from both classical consanguinity and genetic drift. Negative *F*_IS_ values ranging from − 0.0005 ± 0.0192 to -0.0490 ± 0.0333 were detected in all individuals in our study of the WM metapopulation. Interestingly, positive *F*_IS_ values have been observed only in native groups inhabiting areas on the fringe of the Near/Middle East, indicating the encouragement of relative mating as well as a deficiency of heterozygous genotypes. The Bedouin community was distinguished by the highest values of *F*_ROH_ = 0.0438 ± 0.0292 and *F*_IS_ = 0.0343 ± 0.0401. When calculating *F*_IS_ values stratified by continental ancestry groups (Europe, North Africa and Middle East) findings are equivalent to those obtained when analyzing population by population (see Table S4). Both European Mediterranean and North African groups yielded negative *F*_IS_ values (-0.0068 and - 0.01491, respectively). The unexpected excess of heterozygotes among native North Africans could be explained by continental admixture events related to trans-Saharan migrations. In this line, Lucas-Sanchez et al. (2023) identified sub-Saharan-like ancestry segments in the genomes of current-day North Africans from Tunisia and Morocco. In contrast, the Middle East group, which contain Druze, Palestinian and Bedouin population samples, keeps the highest and most positive value of *F*_IS_ (0.01829), indicating that this deficiency of heterozygotes should be interpreted as a clear hallmark of strong consanguineous behavior still prevailing among these communities.

### ROH Islands (ROHi)

Table 2 shows the global number and length of ROHi together with the mean frequency of individuals sharing ROHi within each population. Additionally, the maximum values shared for a single island for the complete population set (*islands_step1*; see Materials and Methods) are depicted. The number of ROHi detected is clearly dependent on the sample size, with the highest values identified both in the IBS and TSI populations. The rest of the parameters are more likely linked to the demographic and consanguineous histories of populations. The lowest maximum sharing values of a single island are found in 1KGP populations (IBS: 58.20%, TSI: 62.14%), presumably encompassing individuals with broader geographic and familial origins.

**Table 2:** ROHi global parameters estimated by population (*islands_step1*). Western Mediterranean populations are highlighted in italics. Length of ROHi is shown in Mb

Populations	*N*	Number of ROHi	Length of ROHi			Percentage of individuals sharing ROHi		Maximum sharing value of a single island
			Mean	SD	Max	Mean	SD	
*E. Andal. (Granada) (SP)*	35	429	0.36	0.41	5.30	44.68	12.13	100.00
*W. Andal. (Huelva) (SP)*	35	420	0.36	0.40	5.30	46.02	12.23	100.00
*South Portugal (PT)*	35	452	0.35	0.36	5.30	44.66	12.33	100.00
Iberia (IB)	99	824	0.35	0.29	2.3	26.03	6.45	58.20
Toscana (IT)	92	788	0.36	0.31	3.20	26.28	6.44	62.14
Sardinia (IT)	28	290	0.31	0.23	1.50	47.30	8.71	92.86
*Asni Berbers (MO)*	15	124	0.40	0.55	5.30	62.85	15.04	100.00
*Bouhria Berbers (MO)*	10	93	0.42	0.62	5.30	73.55	11.48	100.00
*Figuig Berbers (MO)*	9	59	0.43	0.47	2.40	80.06	11.44	100.00
Mozabite Berbers (MO)	27	168	0.28	0.21	1.60	42.16	6.37	64.20
Bedouin (IS)	46	328	0.31	0.26	2.00	40.38	7.18	67.75
Druze (Negel) (IS)	42	343	0.31	0.25	1.70	42.50	7.58	78.57
Palestinian (IS)	46	413	0.32	0.26	3.10	36.73	6.79	67.22

When islands with high representation within populations (≥ 90%) were inspected, the WM region (present study) presented the highest levels of common ROHi both within and among populations (Table S5). For the referenced area, most islands were nonprivate but commonly present across populations, indeed showing full integrity of the island (C-2, C-3, C-4, C-21, and C-22) or overlapping genomic coordinates (C-1, C-7, C-9, C-10, C-14, and C-16). The genomic distribution of ROHi in WM populations is graphically shown in Figure S1. Highly represented islands shared by at least two populations are depicted in orange, mirroring the information displayed in Table S5. Karyograms generated for the rest of the population set are shown in Figure S2.

For WM populations, further gene analyses within ROHi segments revealed an enrichment of candidate genes involved in the adaptive immune response and immunoglobulin production, as underlying biological functions, mainly in the Berber samples. The cumulative genomic length of highly represented ROHi revealed mean levels of 6.17 Mb in southern Iberia and 9.43 Mb in Moroccan Berbers [as estimated from the “Length (Mb)” column of Table S5].

The occurrence of ROHi ≥ 1.5 Mb per population (*islands_step1*) was also explored (Table S6). Interestingly, the longest island (5.3 Mb, C-9) is simultaneously present at high incidence (from 66.70 to 82.90%) in all WM populations except Figuig Berbers. A 2.3-Mb-long ROHi (positions 68300000–70600000, hg19 genome version) that overlaps with the previous ROHi was identified at high levels in Figuig people (88.90%). In addition, long overlapping islands characterize WM genomes in C-16 (mean length: 2.8 Mb, mean incidence: 91.50%) and C-1 (mean length: 2 Mb, mean incidence: 84.38%). These highly conserved ROHi in both chromosomes seem to be enriched in: *(i)* the immunoglobulin-mediated immune response, for C-16 (see Table S6, Supplementary Tables, pp.11–12) and *(ii)* in protein isomerization and folding, for C-1 (see Table S6, Supplementary Tables, p.13).

The ROHi distribution patterns by chromosome curiously showed parallel scenarios to those observed when defining ROH coverage per chromosome (Fig. 4), where C-1, C-9 and C-16 yielded the highest values.

When the metapopulation approach was used (*islands_step2*, see Materials and Methods), the mean values of the shared islands consequently decreased since distinctive populations were merged (Table 3). However, the highest mean (29.44%) and maximum percentages (98.94%) of shared ROHi remained registered in the WM populations. These values are much lower than those reported in the other Mediterranean groups.

**Table 3:** ROHi global parameters estimated in each of the Mediterranean metapopulations (*islands_step2*). Populations considered within each group is described in Material and methods

		Number of ROHi	Length of ROHi			Percentage of individuals sharing ROHi		Maximum sharing value of a single island
	*N*		Mean	SD	Max	Mean	SD	
Western Mediterranean	139	942	0.40	0.40	5.30	29.44	10.31	98.94
Central Mediterranean	120	814	0.37	0.35	4.00	24.89	5.51	56.90
Eastern Mediterranean	134	867	0.36	0.31	3.10	29.27	6.06	66.99

**Fig. 6:**
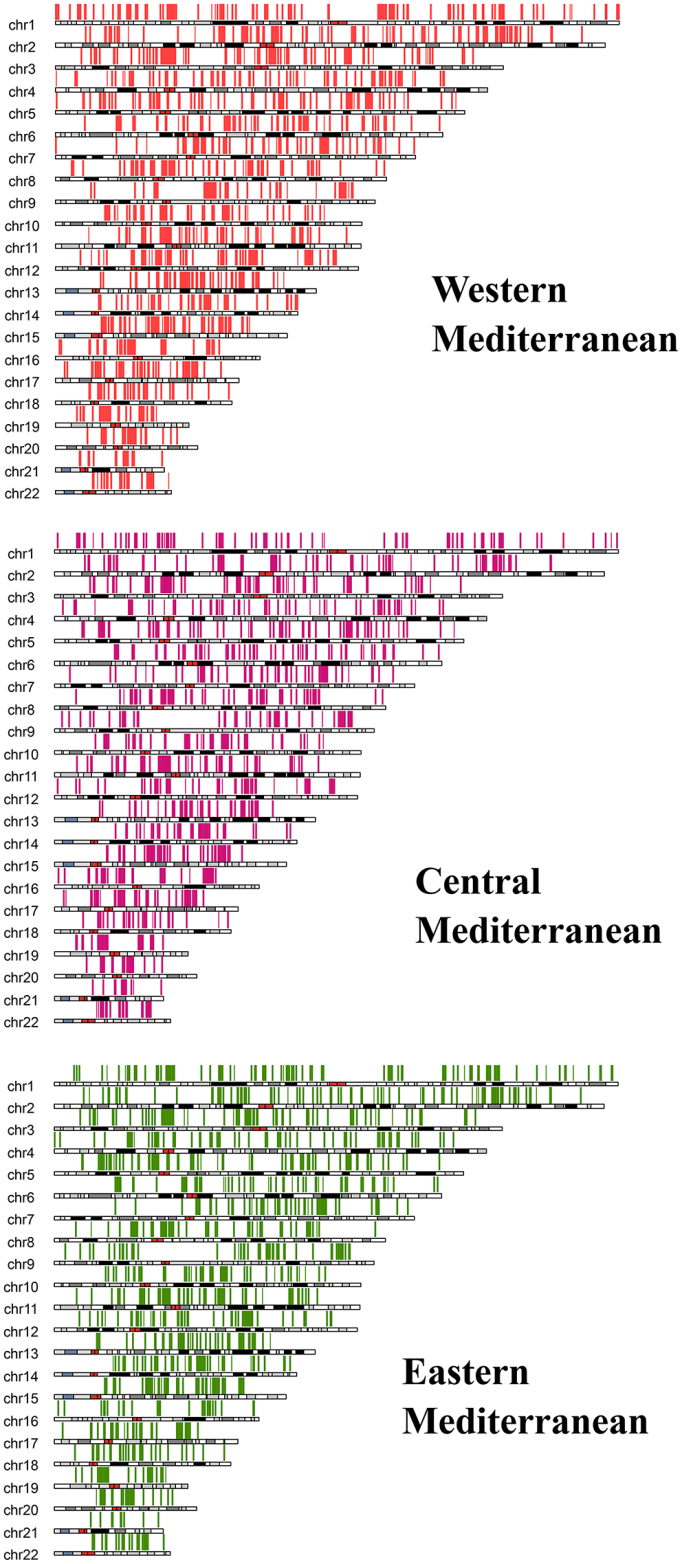
ROHi genomic distribution across in the three metapopulations built (*islands_step2*, see description in Material and methods)

Here, only a single island was shared above the 90% threshold (C-21, coordinates 10700000–11100000, 0.4 Mb), identified in the WM group (data not shown in the tables). Nevertheless, analogous results with respect to *islands_step1* were found when exploring large ROHi values (Table S7). It is worth highlighting the common and exclusive presence of the longest ROHi (5.3 Mb) in C-9 among WM individuals. This observation reveals common patterns of genomic autozygosity across the Strait of Gibraltar (see Figure S3). Likewise, other shared ROHi detected through Mediterranean populations could be coherently interpreted in terms of their genetic relatedness (Figures S4 and S5). Henceforth, seven overlapping ROHi appear simultaneously in the three Mediterranean subregions. When looking at those islands exclusively present by pairs, WM-CM shares five ROHi, and CM-EM shares two ROHi. Interestingly, WM-EM did not record any islands in common. A general view of the genomic distribution of ROHi in the western, central and eastern metapopulations is depicted in Fig. 6.

Finally, some interesting signatures were drawn when inspecting the ROHi detected in other previous studies (Table S8). Some conserved signals of selection can be inferred from the common presence of ROHi in African and European populations around the *LCT* and *MCM6* genes, both of which are involved in lactase persistence (LP) polymorphisms. Another trait with potential evolutionary significance is the sensory perception of smell, which is significantly enriched in the ROHi detected in C-9 and C-11. This signature was found in European populations as a whole (Nothnagel et al. 2010) and in the western Mediterranean (present study) but not in sub-Saharan Africa (Ceballos et al. 2019).

## Discussion

In most human populations, ROH’s architecture mirrors their evolutionary past, as it provides a rich record from which to interpret demographic histories and cultural practices (marital preferences) (Kirin et al. 2010; Lemes et al. 2018; Ceballos et al. 2019; Mezzavilla et al. 2022). In addition, the timing and number of admixture events impact ROH architecture, as the number of ROH in admixed populations decreases as a consequence of the union of two very different ancestral population origins. Parental populations can be distinguished by distinctive ROH profiles, causing a reduction in *F*_ROH_ levels and, thus, an increase in genetic diversity.

The ROH structures in southern Iberians and Moroccan Berbers showed appreciable differences. The high representation in Berbers of the longest ROH segments should be accepted as a distinctive hallmark of their genomic structure. This scenario is consistent with that detected in other human populations characterized by high rates of consanguineous mating and significant levels of inbreeding (Pemberton and Rosenberg 2014; Bergström et al. 2020).

By accepting that shorter ROH account for the highest weight of the total homozygosity of the genome even in most inbred populations (see Kirin et al. 2010; Pemberton et al. 2012; among others), an increase in longer ROH in individual genomes would presumably lead to higher *F*_ROH_ values. Similarly, we found an approximately twofold greater mean value of *F*_ROH_ in Moroccan Berbers than in southern Iberians. Consequently, the impact of greater ROH on *F*_ROH_, as a signature of recent inbreeding due to either genetic drift or consanguinity, should be considered a good predictor of inbreeding levels based on pedigrees, *F*_PED_ (McQuillan et al. 2008; Keller et al. 2011). Nevertheless, this assumption remains controversial.

The magnitude of genome-wide homozygosity inferred from ROH (*F*_ROH_) seems to be much greater than that inferred from genealogical individual inbreeding coefficients based on three- or four-generation (*F*_PED_) [i.e., up to second (M33) or third cousin (M44) unions]. For example, among mainland Spanish populations, the mean inbreeding coefficient, *F*_PED_, of the general population, which is based on Catholic Church ecclesiastical dispensations, is approximately 0.00270 on average. The high number of published surveys covered a wide temporal range (mostly 1900 to 1979) (Calderón et al. 2009, 2018).

In addition to cultural (mating patterns) and demographic factors, other variables that can shape ROH profiles constitute the natural selection process. Given the complexity and density of ROH fragments across the genome of WM populations, we moved to the broader picture that ROH island analysis could provide. The presence of ROHi has been studied in scattered publications (Curtis et al. 2008; Nothnagel et al. 2010; Pemberton et al. 2012; Ceballos et al. 2019). The findings presented therein reflect inherent heterogeneities among human populations. The maximum sharing values of ROHi record values ranging from 30 to 68% in populations of European descent (Curtis et al. 2008). Proportions in African groups rank between 20% and 36%. These results are rather small compared with some of those obtained in the present study, where some ROHi are present in all individuals (100%) (Table 2) as a signature of internal local links that are not observed within Central and Eastern Mediterranean populations.

Compared with the other genomes, the Moroccan Berber genomes are characterized by the longest ROHi (0.40–0.43 Mb). Moreover, ROHi are more frequently shared among individuals within populations (the mean value of individuals sharing islands spans from 62.85% in Asni to 80.06% in Figuig). However, in southern Iberians, the proportions are more modest (global mean 45.12%). Notably, the highest frequencies (100%) of sharing islands were found among the members of the studied WM population samples. This means that at least one single ROHi is present in all the members belonging to a certain population.

The presence of shared ROHi seems to be dependent on geography and could reflect common past histories. In this context, WM people contain more islands in common with CM people than with their more distant relatives from the eastern side. This scenario constitutes an example of an isolation-by-distance pattern. Different interpretations have been proposed for explaining ROHi locations: *i*) low-recombination regions (Curtis et al. 2008), *ii*) regions containing few deleterious recessive alleles, and *iii*) potential targets of selective sweeps (Pemberton et al. 2012). With respect to the first mechanism, we indeed observed a high concentration of ROHi near the centromeres, although this hypothesis does not explain the complete scenario.

The interpretation of ROHi as a signal of positive selection has been extensively addressed by Pemberton et al. (2012). The authors reported differences in ROH hotspot locations between human continental groups, highlighting the relevant biological functions of some genes [e.g., human pigmentation (*KITLG*)] contained in ROHi reported in non-Africans. Certain ROHi detected in the present survey replicate previous results that have been interpreted in selective terms; for example, the lactase gene (*LCT*) and its surrounding *MCM6* locus are located (contained) within a single ROHi (Nothnagel et al. 2010).

Our results also underline two gene ontology (GO) terms significantly enriched in ROHi with relevant evolutionary implications: *i*) immune response, including immunoglobulin genes (*IGKV*, *IGH* and *IGL* clusters) whose signals are dispersed across the genome. These genes have been previously reported as targets of positive selection (Vallender and Lahn 2004; Walsh et al. 2020); and *ii*) sensory perception of smell [e.g., vomeronasal receptor genes (*VN1R*)] or genes encoding olfactory receptors (*ORs*, families 1, 2, 4, 5, 11, 12 and 14). Chemosensory genes have been studied for their connection of local adaptation and human subsistence behavior to natural selection (Veilleux et al. 2023).

As mentioned above, the most relevant autozygous hotspot block (in terms of size and representation), a 5.3-Mb-long single island in C-9, was found only in WM individuals (present work) and was absent in the remaining Mediterranean samples analyzed. To the best of our knowledge, this is the longest ROHi reported in the literature. The segment, with no GO enrichment detected, harbors members of the cytochrome *P450* family genes and miosine *VB* coding genes, although it is mostly composed of pseudogenes that could be in linkage disequilibrium with adjacent, clearer targets of positive selection (Kelley et al. 2006). However, while pseudogenes are considered to escape the constraints of selection, given their noncoding characteristic (Torrents et al. 2003), some of them could be considered a genomic reservoir with a somehow biological function (Zhang and Zheng 2014). Given this condition, we cannot rule out the possibility that selective processes could have been involved in the conservation of this specific ROHi across the Strait of Gibraltar.

Overall, the ROHi results, that is, the consistent sharing of islands, provide further support for the definition of the concept of a *metapopulation* in the WM area. The complexity of human relationships between populations living around the Strait of Gibraltar has been studied through the use of different genomic markers with different molecular architectures. Uniparental markers and, specifically, maternal U6, M1 and L, and paternal E-M81 African lineages, together with maternal haplogroup H and paternal R-M269 European markers, have provided substantial information (Coudray et al. 2009; Cerezo et al. 2012; Bekada et al. 2013; and other references therein) about ancient population movements at the extreme edge of the Mediterranean. One of the most interesting conclusions is the concentration of African contributions across the Iberian Atlantic façade, with the highest values observed in the westernmost end of the Andalusia region (U6 maternal lineage, 7.5% mtDNA diversity; Hernández et al. 2015) and in southern Portugal (E-M81 paternal lineage, 12.2%; Cruciani et al. 2004). Nevertheless, the latter and more decisive evidence, observed in the same populations studied here, on the basis of genome-wide (GW) data, revealed that the highest traces of the North African and sub-Saharan ancestral components are found in southern Portugal (21% of its estimated global ancestry, ADMIXTURE approach). In contrast, the Berber population of Bouhria harbors the highest estimate of European ancestry within the Maghreb (20%, ADMIXTURE data) (see Hernández et al. 2020).

The intertwined evolutionary history of WM populations has been inferred not only from genomic data but also from cultural and archaeological evidence (Hernández and Calderón 2017). Studies based on ancient DNA (aDNA) are being conducted to understand the evolution of cultural practices that have encouraged consanguineous unions in the most recent past of our species. Notably, both Upper Paleolithic and Mesolithic hunter–gatherer groups (~ 12000 − 6000 cal BC) of different continental origins carried high levels of ROH in their genomes. Ringbauer et al. (2021), analyzing DNA samples of individuals who lived in the last 45,000 years, reported a decline in parental relatedness coinciding with the presence of the first Neolithic farmer settlements. These genome-wide results are supported by the great changes that occurred during the Neolithic transition, a fundamental period where new sedentary and agricultural Neolithic lifestyles were accompanied by major demographic shifts toward increased local population sizes and admixture histories. This sustained progression culminated in Bronze Age populations characterized by low ROH levels, similar to modern Europeans (Ceballos et al. 2021).

Today, there is fair recognition of the importance of studying human consanguinity from genomic data, irrespective of the availability of historical ecclesiastical registers or pedigree information. These new approaches represent a key resource for addressing relevant questions for anthropological and human genetics. Genomic inbreeding estimates (autozygosity levels) provide an open window into the understanding of marital patterns, kinship analysis, sociocultural behaviors and demographic histories of past and present-day human populations. The relationships between autozygosity and disease risk, reproductive success and other traits represent a matter of great interest for biomedicine.

In the frame of the WM, consanguinity rates and structure, chronological variation in the types of consanguineous marriages and mean inbreeding coefficients, based on historical registers (i.e., Catholic Church dispensations), have been thoroughly analyzed in many populations with varying population sizes from the Iberian Peninsula, mostly from Spain. Consanguinity in Spain has been secularly high, with significant geographic variations, and its decline has been faster and later than that in other European countries (Calderón et al. 1993). Nevertheless, modern studies on ROH architecture, genomic inbreeding levels and their epidemiological consequences, particularly in Spanish regions and populations, are almost nonexistent. Thus, new efforts and initiatives to address Iberian populations in accordance with these advanced molecular and population genetic approaches are desirable.

## Conclusion

The screening of autozygous segments in human genomes from the western Mediterranean (Iberia and Morocco) reveals insights into past demographic dynamics and genetic relationships of the people settled at the crossroads of Europe and Africa. Furthermore, gene enrichment signals drawn from ROH fragments allow us to deepen our understanding of the evolutionary mechanisms that have driven recent population histories.

## Electronic supplementary material

Below is the link to the electronic supplementary material.

Supplementary Material 1

Supplementary Material 2

## Data Availability

No new data was created in this study. Raw genomic data from Hernández et al. 2020 is available at the EGA repository (European Genome-Phenome Archive; Study Accession Number: EGAS00001003901).

## References

[CR1] Alfonso-Sánchez M, Calderón R, Peña J (2004) Opportunity for natural selection in a Basque population and its secular trend: evolutionary implications of epidemic mortality. Hum Biol 76:361–381. 10.1353/hub.2004.0041 15481673

[CR2] Alvarez G, Ceballos FC, Quinteiro C (2009) The role of inbreeding in the extinction of a European Royal dynasty. PLoS ONE 4:1–7. 10.1371/journal.pone.0005174 10.1371/journal.pone.0005174 PMC2664480 19367331

[CR3] Ambrosio B, Hernández C, Novelletto A et al (2010) Searching the peopling of the Iberian Peninsula from the perspective of two Andalusian subpopulations: A study based on Y-chromosome haplogroups J and E. Coll Antropol 34:1215–1228 21874703

[CR4] Auton A, Bryc K, Boyko AR et al (2009) Global distribution of genomic diversity underscores rich complex history of continental human populations. Genome Res 19:795–803. 10.1101/gr.088898.108 19218534 10.1101/gr.088898.108 PMC2675968

[CR5] Auton A, Abecasis GR, Altshuler DM et al (2015) A global reference for human genetic variation. Nature 526:68–74. 10.1038/nature15393 26432245 10.1038/nature15393 PMC4750478

[CR6] Bacolod MD, Schemmann GS, Wang S et al (2008) The signatures of autozygosity among patients with colorectal Cancer. Cancer Res 68:2610–2621. 10.1158/0008-5472.CAN-07-5250.The 18375840 10.1158/0008-5472.CAN-07-5250 PMC4383032

[CR7] Bekada A, Fregel R, Cabrera VM et al (2013) Introducing the Algerian mitochondrial DNA and Y-chromosome profiles into the North African landscape. PLoS ONE 8:e56775. 10.1371/journal.pone.0056775 23431392 10.1371/journal.pone.0056775 PMC3576335

[CR8] Bergström A, McCarthy SA, Hui R et al (2020) Insights into human genetic variation and population history from 929 diverse genomes. Science 367:eaay5012. 10.1126/science.aay5012 32193295 10.1126/science.aay5012 PMC7115999

[CR11] Bittles AH, Black ML (2010) Consanguinity, human evolution, and complex diseases. Proc Natl Acad Sci U S A 107:1779–1786. 10.1073/pnas.0906079106 19805052 10.1073/pnas.0906079106 PMC2868287

[CR9] Bittles AH, Neel JV (1994) The costs of human inbreeding and their implications for variations at the DNA level. Nat Genet 8:117–121. 10.1038/ng1094-117 7842008 10.1038/ng1094-117

[CR10] Bittles A, Wang W, Savithri H et al (2001) Human inbreeding: a familiar story full of surprises. In: Macbeth E, Shetty P (eds) Health and ethnicity. CRC, London, pp 68–78

[CR12] Calderon R (1989) Consanguinity in the archbishopric of Toledo, Spain, 1900-79. I. Types of consanguineous mating in relation to premarital migration and its effects on inbreeding levels. J Biosoc Sci 21:253–266. 10.1017/S002193200001796X 2670948 10.1017/s002193200001796x

[CR13] Calderón R, Peña JA, Morales B, Guevara JI (1993) Inbreeding patterns in the Basque country (Alava Province, 1831–1980). Hum Biol 65:743–770 8262504

[CR14] Calderón R, Aresti U, Ambrosio B, Rosa JM (2005) Geographic, demographic and inbreeding patterns in a Basque mountainous region of Guipuzcoa. Int J Anthropol 20:173–197. 10.1007/BF02443057

[CR15] Calderón R, Ambrosio B, Guitard E et al (2006) Genetic position of Andalusians from Huelva in relation to other European and North African populations: a study based on GM and KM allotypes. Hum Biol 78:663–679 17564246 10.1353/hub.2007.0008

[CR16] Calderón R, Aresti U, Ambrosio B, González-Martín A (2009) Inbreeding coefficients for X-linked and autosomal genes in consanguineous marriages in Spanish populations: the case of Guipúzcoa (Basque Country). Ann Hum Genet 73:184–195. 10.1111/j.1469-1809.2008.00495.x 19133940 10.1111/j.1469-1809.2008.00495.x

[CR17] Calderón R, Hernández CL, García-Varela G et al (2018) Inbreeding in southeastern Spain. The impact of geography and demography on marital mobility and marital distance patterns (1900–1969). Hum Nat 29:45–64. 10.1007/s12110-017-9305-z 29159722 10.1007/s12110-017-9305-z

[CR18] Cavalli-Sforza LL, Bodmer WF (1971) The genetics of human populations. W. H. Freeman and Company, San Francisco

[CR19] Cavalli-Sforza LL, Moroni A, Zei G (2004) Consanguinity, inbreeding and genetic drift in Italy. Princeton University Press, Oxford, UK

[CR20] Ceballos FC, Joshi PK, Clark DW et al (2018) Runs of homozygosity: windows into population history and trait architecture. Nat Rev Genet 19:220–234. 10.1038/nrg.2017.109 29335644 10.1038/nrg.2017.109

[CR21] Ceballos FC, Hazelhurst S, Ramsay M (2019) Runs of homozygosity in sub-Saharan African populations provide insights into complex demographic histories. Hum Genet 138:1123–1142. 10.1007/s00439-019-02045-1 31312899 10.1007/s00439-019-02045-1

[CR22] Ceballos FC, Gürün K, Altınışık NE et al (2021) Human inbreeding has decreased in time through the holocene. Curr Biol 31:3925–3934e8. 10.1016/j.cub.2021.06.027 34216555 10.1016/j.cub.2021.06.027

[CR23] Cerezo M, Achilli A, Olivieri A et al (2012) Reconstructing ancient mitochondrial DNA links between Africa and Europe. Genome Res 22:821–826. 10.1101/gr.134452.111 22454235 10.1101/gr.134452.111 PMC3337428

[CR24] Chang CC, Chow CC, Tellier LCAM et al (2015) Second-generation PLINK: rising to the challenge of larger and richer datasets. Gigascience 4:1–16. 10.1186/s13742-015-0047-8 25722852 10.1186/s13742-015-0047-8 PMC4342193

[CR25] Christianson A, Howson C, Modell B (2006) Global report on birth defects. March of dimes global report on birth defects. White Plains, New York, pp 83–84

[CR26] Conomos MP, Reiner AP, Weir BS, Thornton TA (2016) Model-free Estimation of recent genetic relatedness. Am J Hum Genet 98:127–148. 10.1016/j.ajhg.2015.11.022 26748516 10.1016/j.ajhg.2015.11.022 PMC4716688

[CR27] Coudray C, Olivieri A, Achilli A et al (2009) The complex and diversified mitochondrial gene pool of Berber populations. Ann Hum Genet 73:196–214. 10.1111/j.1469-1809.2008.00493.x 19053990 10.1111/j.1469-1809.2008.00493.x

[CR28] Cruciani F, La Fratta R, Santolamazza P et al (2004) Phylogeographic analysis of haplogroup E3b (E-M215) y chromosomes reveals multiple migratory events within and out of Africa. Am J Hum Genet 74:1014–1022. 10.1086/386294 15042509 10.1086/386294 PMC1181964

[CR29] Curtis D, Vine AE, Knight J (2008) Study of regions of extended homozygosity provides a powerful method to explore haplotype structure of human populations. Ann Hum Genet 72:261–278. 10.1111/j.1469-1809.2007.00411.x 18205893 10.1111/j.1469-1809.2007.00411.x PMC2343471

[CR30] Durinck S, Spellman PT, Birney E, Huber W (2009) Mapping identifiers for the integration of genomic datasets with the R/Bioconductor package biomart. Nat Protoc 4:1184–1191. 10.1038/nprot.2009.97 19617889 10.1038/nprot.2009.97 PMC3159387

[CR31] Elliott KS, Haber M, Daggag H et al (2022) Fine-Scale genetic structure in the united Arab Emirates reflects endogamous and consanguineous culture, population history, and geography. Mol Biol Evol 39:1–11. 10.1093/molbev/msac039 10.1093/molbev/msac039 PMC8911814 35192718

[CR32] Ferreira JC, Alshamali F, Pereira L, Fernandes V (2022) Characterization of Arabian Peninsula whole exomes: contributing to the catalogue of human diversity. iScience 25:105336. 10.1016/j.isci.2022.105336 36325056 10.1016/j.isci.2022.105336 PMC9619305

[CR33] Frazer KA, Ballinger DG, Cox DR et al (2007) A second generation human haplotype map of over 3.1 million SNPs. Nature 449:851–861. 10.1038/nature06258 17943122 10.1038/nature06258 PMC2689609

[CR34] Gazal S, Sahbatou M, Perdry H et al (2014) Inbreeding coefficient Estimation with dense SNP data: comparison of strategies and application to HapMap III. Hum Hered 77:49–62. 10.1159/000358224 25060269 10.1159/000358224

[CR35] Ghani M, Reitz C, Cheng R et al (2015) Association of long runs of homozygosity with alzheimer disease among African American individuals. JAMA Neurol 72:1313–1323. 10.1001/jamaneurol.2015.1700 26366463 10.1001/jamaneurol.2015.1700 PMC4641052

[CR36] Giacopuzzi E, Gennarelli M, Minelli A et al (2017) Exome sequencing in schizophrenic patients with high levels of homozygosity identifies novel and extremely rare mutations in the GABA/glutamatergic pathways. PLoS ONE 12:1–12. 10.1371/journal.pone.0182778 10.1371/journal.pone.0182778 PMC5546675 28787007

[CR37] Gibson J, Morton NE, Collins A (2006) Extended tracts of homozygosity in outbred human populations. Hum Mol Genet 15:789–795. 10.1093/hmg/ddi493 16436455 10.1093/hmg/ddi493

[CR38] Gorssen W, Meyermans R, Janssens S, Buys N (2021) A publicly available repository of ROH Islands reveals signatures of selection in different livestock and pet species. Genet Sel Evol 53:1–10. 10.1186/s12711-020-00599-7 33397285 10.1186/s12711-020-00599-7 PMC7784028

[CR39] Grilz-Seger G, Druml T, Neuditschko M et al (2019) Analysis of ROH patterns in the Noriker horse breed reveals signatures of selection for coat color and body size. Anim Genet 50:334–346. 10.1111/age.12797 31199540 10.1111/age.12797 PMC6617995

[CR40] Hamamy H, Antonarakis SE, Cavalli-Sforza LL et al (2011) Consanguineous marriages, pearls and perils: Geneva international consanguinity workshop report. Genet Med 13:841–847. 10.1097/GIM.0b013e318217477f 21555946 10.1097/GIM.0b013e318217477f

[CR41] Hartl D, Clark A (2006) Principles of population genetics. Sinauer Associates, Sunderland, Massachusetts

[CR42] Hedrick PW (2005) Genetics of populations. Jones and Bartlett publishers. Sudbury, Masachusetts

[CR44] Hernández CL, Calderón R (2017) Matrilineal heritage in Southern Iberia reveals deep genetic links between continents. Coll Antropol 41:1–10 29139642

[CR43] Hernández CL, Soares P, Dugoujon JM et al (2015) Early holocenic and historic MtDNA African signatures in the Iberian Peninsula: the Andalusian region as a paradigm. PLoS ONE 10:e0139784. 10.1371/journal.pone.0139784 26509580 10.1371/journal.pone.0139784 PMC4624789

[CR45] Hernández CL, Pita G, Cavadas B et al (2020) Human genomic diversity where the mediterranean joins the Atlantic. Mol Biol Evol 37:1041–1055. 10.1093/molbev/msz288 31816048 10.1093/molbev/msz288 PMC7086172

[CR46] Kang JTL, Goldberg A, Edge MD et al (2016) Consanguinity rates predict long runs of homozygosity in Jewish populations. Hum Hered 82:87–102. 10.1159/000478897 28910803 10.1159/000478897 PMC5698150

[CR47] Karafet TM, Bulayeva KB, Bulayev OA et al (2015) Extensive genome-wide autozygosity in the population isolates of Daghestan. Eur J Hum Genet 23:1405–1412. 10.1038/ejhg.2014.299 25604856 10.1038/ejhg.2014.299 PMC4592092

[CR48] Keller MC, Visscher PM, Goddard ME (2011) Quantification of inbreeding due to distant ancestors and its detection using dense single nucleotide polymorphism data. Genetics 189:237–249. 10.1534/genetics.111.130922 21705750 10.1534/genetics.111.130922 PMC3176119

[CR49] Keller MC, Simonson MA, Ripke S et al (2012) Runs of homozygosity implicate autozygosity as a schizophrenia risk factor. PLoS Genet 8:1–11. 10.1371/journal.pgen.1002656 10.1371/journal.pgen.1002656 PMC3325203 22511889

[CR50] Kelley JL, Madeoy J, Calhoun JC et al (2006) Genomic signatures of positive selection in humans and the limits of outlier approaches. Genome Res 16:980–989. 10.1101/gr.5157306 16825663 10.1101/gr.5157306 PMC1524870

[CR51] Kirin M, McQuillan R, Franklin CS et al (2010) Genomic runs of homozygosity record population history and consanguinity. PLoS ONE 5:e13996. 10.1371/journal.pone.0013996 21085596 10.1371/journal.pone.0013996 PMC2981575

[CR52] Lasker G (1985) Surnames and genetic structure. Cambridge University Press, Cambridge

[CR53] Lasker GW (1988) Application of surname frequency distributions to studies of mating preference. In: Mascie-Taylor CGN, Boyce AJ (eds) Human mating patterns. Cambridge University Press, Cambridge

[CR54] Lemes RB, Nunes K, Carnavalli JEP et al (2018) Inbreeding estimates in human populations: applying new approaches to an admixed Brazilian isolate. PLoS ONE 13:1–14. 10.1371/journal.pone.0196360 10.1371/journal.pone.0196360 PMC5916862 29689090

[CR55] Lencz T, Lambert C, DeRosse P et al (2007) Runs of homozygosity reveal highly penetrant recessive loci in schizophrenia. Proc Natl Acad Sci U S A 104:19942–19947. 10.1073/pnas.0710021104 18077426 10.1073/pnas.0710021104 PMC2148402

[CR56] Li L-H, Ho S-F, Chen C-H et al (2006) Long contiguous stretches of homozygosity in the human genome. Hum Mutat 27:1115–1121. 10.1002/humu.20399 16955415 10.1002/humu.20399

[CR57] Lucas-Sánchez M, Fadhlaoui-Zid K, Comas D (2023) The genomic analysis of current-day North African populations reveals the existence of trans-Saharan migrations with different origins and dates. Hum Genet 142:305–320. 10.1007/s00439-022-02503-3 36441222 10.1007/s00439-022-02503-3 PMC9918576

[CR58] Malawsky DS, van Walree E, Jacobs BM et al (2023) Influence of autozygosity on common disease risk across the phenotypic spectrum. Cell 186:4514–4527e14. 10.1016/j.cell.2023.08.028 37757828 10.1016/j.cell.2023.08.028 PMC10580289

[CR59] Mastrangelo S, Sardina MT, Tolone M et al (2018) Genome-wide identification of runs of homozygosity Islands and associated genes in local dairy cattle breeds. Animal 12:2480–2488. 10.1017/S1751731118000629 29576040 10.1017/S1751731118000629

[CR60] McQuillan R, Leutenegger A-L, Abdel-Rahman R et al (2008) Runs of homozygosity in European populations. Am J Hum Genet 83:359–372. 10.1016/j.ajhg.2008.08.007 18760389 10.1016/j.ajhg.2008.08.007 PMC2556426

[CR61] Meyermans R, Gorssen W, Buys N, Janssens S (2020) How to study runs of homozygosity using plink? A guide for analyzing medium density Snp data in livestock and pet species. BMC Genomics 21:1–14. 10.1186/s12864-020-6463-x 10.1186/s12864-020-6463-x PMC6990544 31996125

[CR62] Mezzavilla M, Cocca M, Maisano Delser P et al (2022) Ancestry-related distribution of runs of homozygosity and functional variants in Qatari population. BMC Genomic Data 23:1–10. 10.1186/s12863-022-01087-1 36131251 10.1186/s12863-022-01087-1 PMC9490902

[CR63] Modell B, Darr A (2002) Genetic counselling and customary consanguineous marriage. Nat Rev Genet 3:225–229. 10.1038/nrg754 11972160 10.1038/nrg754

[CR64] Moreno-Grau S, Fernández MV, de Rojas I et al (2021) Long runs of homozygosity are associated with Alzheimer’s disease. Transl Psychiatry 11:1–13. 10.1038/s41398-020-01145-1 33627629 10.1038/s41398-020-01145-1 PMC7904832

[CR65] Moroni A, Amelli A, Anguinetti W et al (1972) La consanguineita Umana Nell’isola Di Sardegna Dal Secolo XVIII al Secolo XX. Estratto dall’Ateneo Parm 8:69–92

[CR66] Nothnagel M, Lu TT, Kayser M, Krawczak M (2010) Genomic and geographic distribution of SNP-defined runs of homozygosity in Europeans. Hum Mol Genet 19:2927–2935. 10.1093/hmg/ddq198 20462934 10.1093/hmg/ddq198

[CR68] Pemberton TJ, Rosenberg NA (2014) Population-genetic influences on genomic estimates of the inbreeding coefficient: A global perspective. Hum Hered 77:37–48. 10.1159/000362878 25060268 10.1159/000362878 PMC4154368

[CR67] Pemberton TJ, Absher D, Feldman MW et al (2012) Genomic patterns of homozygosity in worldwide human populations. Am J Hum Genet 91:275–292. 10.1016/j.ajhg.2012.06.014 22883143 10.1016/j.ajhg.2012.06.014 PMC3415543

[CR69] Pereira L, Cunha C, Alves C, Amorim A (2005) African female heritage in Iberia: a reassessment of MtDNA lineage distribution in present times. Hum Biol 77:213–229 16201138 10.1353/hub.2005.0041

[CR70] Peripolli E, Munari DP, Silva MVGB et al (2017) Runs of homozygosity: current knowledge and applications in livestock. Anim Genet 48:255–271. 10.1111/age.12526 27910110 10.1111/age.12526

[CR71] Peripolli E, Stafuzza NB, Munari DP et al (2018) Assessment of runs of homozygosity Islands and estimates of genomic inbreeding in Gyr (Bos indicus) dairy cattle. BMC Genomics 19:1–13. 10.1186/s12864-017-4365-3 29316879 10.1186/s12864-017-4365-3 PMC5759835

[CR72] Pettener D (1985) Consanguineous marriages in the upper Bologna appennine (1565–1980): microgeographic variations, pedigree structure and correlation of inbreeding secular trend with changes in population size. Hum Biol 57:267–288 3888814

[CR73] Pinheiro J, Bates D, Team RC (2023) nlme: Linear and Nonlinear Mixed Effects Models

[CR74] Ringbauer H, Novembre J, Steinrücken M (2021) Parental relatedness through time revealed by runs of homozygosity in ancient DNA. Nat Commun 12:1–11. 10.1038/s41467-021-25289-w 34521843 10.1038/s41467-021-25289-w PMC8440622

[CR75] Romdhane L, Mezzi N, Hamdi Y et al (2019) Consanguinity and inbreeding in health and disease in North African populations. Annu Rev Genomics Hum Genet 20:155–179. 10.1146/annurev-genom-083118-014954 31039041 10.1146/annurev-genom-083118-014954

[CR76] Romeo G, Bittles AH (2014) Consanguinity in the contemporary world. Hum Hered 77:6–9. 10.1159/000363352 25060264 10.1159/000363352

[CR77] Simón-Sánchez J, Kilarski LL, Nalls MA et al (2012) Cooperative genome-wide analysis shows increased homozygosity in early onset Parkinson’s disease. PLoS ONE 7:e28787. 10.1371/journal.pone.0028787 22427796 10.1371/journal.pone.0028787 PMC3299635

[CR78] Skourtanioti E, Ringbauer H, Gnecchi Ruscone GA et al (2023) Ancient DNA reveals admixture history and endogamy in the prehistoric Aegean. Nat Ecol Evol 7:290–303. 10.1038/s41559-022-01952-3 36646948 10.1038/s41559-022-01952-3 PMC9911347

[CR79] Slate J, David P, Dodds KG et al (2004) Understanding the relationship between the inbreeding coefficient and multilocus heterozygosity: theoretical expectations and empirical data. Heredity (Edinb) 93:255–265. 10.1038/sj.hdy.6800485 15254488 10.1038/sj.hdy.6800485

[CR80] Stoffel MA, Johnston SE, Pilkington JG, Pemberton JM (2021) Genetic architecture and lifetime dynamics of inbreeding depression in a wild mammal. Nat Commun 12:1–10. 10.1038/s41467-021-23222-9 34016997 10.1038/s41467-021-23222-9 PMC8138023

[CR81] Tadmouri GO, Nair P, Obeid T et al (2009) Consanguinity and reproductive health among Arabs. Reprod Health 6:17. 10.1186/1742-4755-6-17 19811666 10.1186/1742-4755-6-17 PMC2765422

[CR82] Thomsen H, Inacio da Silva Filho M, Fuchs M et al (2016) Evidence of inbreeding in hodgkin lymphoma. PLoS ONE 11:e0154259. 10.1371/journal.pone.0154259 27123581 10.1371/journal.pone.0154259 PMC4849743

[CR83] Torrents D, Suyama M, Zdobnov E, Bork P (2003) A genome-wide survey of human pseudogenes. Genome Res 13:2559–2567. 10.1101/gr.1455503 14656963 10.1101/gr.1455503 PMC403797

[CR84] Vallender EJ, Lahn BT (2004) Positive selection on the human genome. Hum Mol Genet 13: Spec No 2:R245–R254. 10.1093/hmg/ddh253 10.1093/hmg/ddh253 15358731

[CR85] VanRaden PM, Olson KM, Wiggans GR et al (2011) Genomic inbreeding and relationships among Holsteins, jerseys, and brown Swiss. J Dairy Sci 94:5673–5682. 10.3168/jds.2011-4500 22032391 10.3168/jds.2011-4500

[CR86] Veilleux CC, Garrett EC, Pajic P et al (2023) Human subsistence and signatures of selection on chemosensory genes. Commun Biol 6:1–12. 10.1038/s42003-023-05047-y 37400713 10.1038/s42003-023-05047-y PMC10317983

[CR87] Walsh S, Pagani L, Xue Y et al (2020) Positive selection in admixed populations from Ethiopia. BMC Genet 21:1–17. 10.1186/s12863-020-00908-5 33092534 10.1186/s12863-020-00908-5 PMC7580818

[CR88] Wang C, Xu Z, Jin G et al (2013) Genome-wide analysis of runs of homozygosity identifies new susceptibility regions of lung cancer in Han Chinese. J Biomed Res 27:208–214. 10.7555/JBR.27.20130017 23720676 10.7555/JBR.27.20130017 PMC3664727

[CR89] Weiss V (1980) Inbreeding and genetic distance between hierarchically structured populations measured by surname frequencies. Mank Q 21:135–149

[CR90] Wright S (1922) Coefficients of inbreeding and relationship. Am Nat 56:330–338

[CR91] Zhang Z, Zheng D (2014) Pseudogene Evolution in the Human Genome. eLS

