# Correction: A genomic tale of inbreeding in western Mediterranean human populations

**DOI:** 10.1007/s00439-025-02773-7

**Published:** 2025-09-23

**Authors:** Candela L. Hernández, Luis J. Sánchez-Martínez, Francisco C. Ceballos, Jean M. Dugoujon, Luisa Pereira, Rosario Calderón

**Affiliations:** 1 Departamento de Biodiversidad, Ecología y Evolución, Facultad de Ciencias Biológicas, Universidad Complutense de Madrid, Madrid, Spain; 2 Instituto de Salud Carlos III, Santiago de Compostela, 15706 Spain; 3 CNRS UMR 5288 Laboratoire d’Anthropologie Moléculaire et d’Imagerie de Synthèse (AMIS), Université Paul Sabatier Toulouse III, Toulouse, France; 4 i3S, Instituto de Investigação e Inovaçãao em Saúde, Universidade do Porto, Porto, Portugal; 5 Ipatimup, Instituto de Patologia e Imunologia Molecular da Universidade do Porto, Porto, Portugal

**Correction: Human Genetics (2025) 144:615–631** 10.1007/s00439-025-02747-9

The article “A genomic tale of inbreeding in western Mediterranean human populations”, written by Candela L. Hernández, Luis J. Sánchez-Martínez, Francisco C. Ceballos, Jean M. Dugoujon, Luisa Pereira, Rosario Calderón., was originally published under exclusive license to The Author(s), under exclusive licence to Springer-Verlag GmbH Germany, part of Springer Nature. As a result of the subsequent decision to publish the article under the open access model, the article’s copyright notice was changed on 7 August 2025 to © The Author(s) 2025 and the article is now distributed under a Creative Commons Attribution 4.0 International License, which permits use, sharing, adaptation, distribution and reproduction in any medium or format, as long as you give appropriate credit to the original author(s) and the source, provide a link to the Creative Commons licence, and indicate if changes were made. The images or other third party material in this article are included in the article’s Creative Commons licence, unless indicated otherwise in a credit line to the material. If material is not included in the article’s Creative Commons licence and your intended use is not permitted by statutory regulation or exceeds the permitted use, you will need to obtain permission directly from the copyright holder. To view a copy of this licence, visit http://creativecommons.org/licenses/by/4.0.

Open Access funded by: Open Access funding provided thanks to the CRUE-CSIC agreement with Springer Nature.

The Original article has been corrected.

