# Supplementary material for: A genomic tale of inbreeding in western Mediterranean human populations
Source: Hum Genet. 2025 May 10;144(6):615–31. doi: 10.1007/s00439-025-02747-9 (PMC12401773; doi:10.1007/s00439-025-02747-9)
Supplement: Supplementary file 1 — Supplementary Material 1 [file 439_2025_2747_MOESM1_ESM.pdf]

*Supplementary Figures*

# A genomic tale of inbreeding in western Mediterranean human populations

Candela L. Hernández, Luis J. Sánchez-Martínez, Francisco C. Ceballos, Jean-Michel Dugoujon, Luisa Pereira, Rosario Calderón

**Figure S1.** ROHi karyograms in western Mediterranean populations (*islands_step1*). ............... 2

**Figure S2.** ROHi karyograms in populations from literature (*islands_step1*)............................. 3

**Figure S3.** ROHi >1.5Mb in the western Mediterranean (*islands_step2*)................................... 4

**Figure S4.** ROHi >1.5Mb in the central Mediterranean (*islands_step2*)..................................... 5

**Figure S5.** ROHi >1.5Mb in the eastern Mediterranean (*islands_step2*). .................................. 6

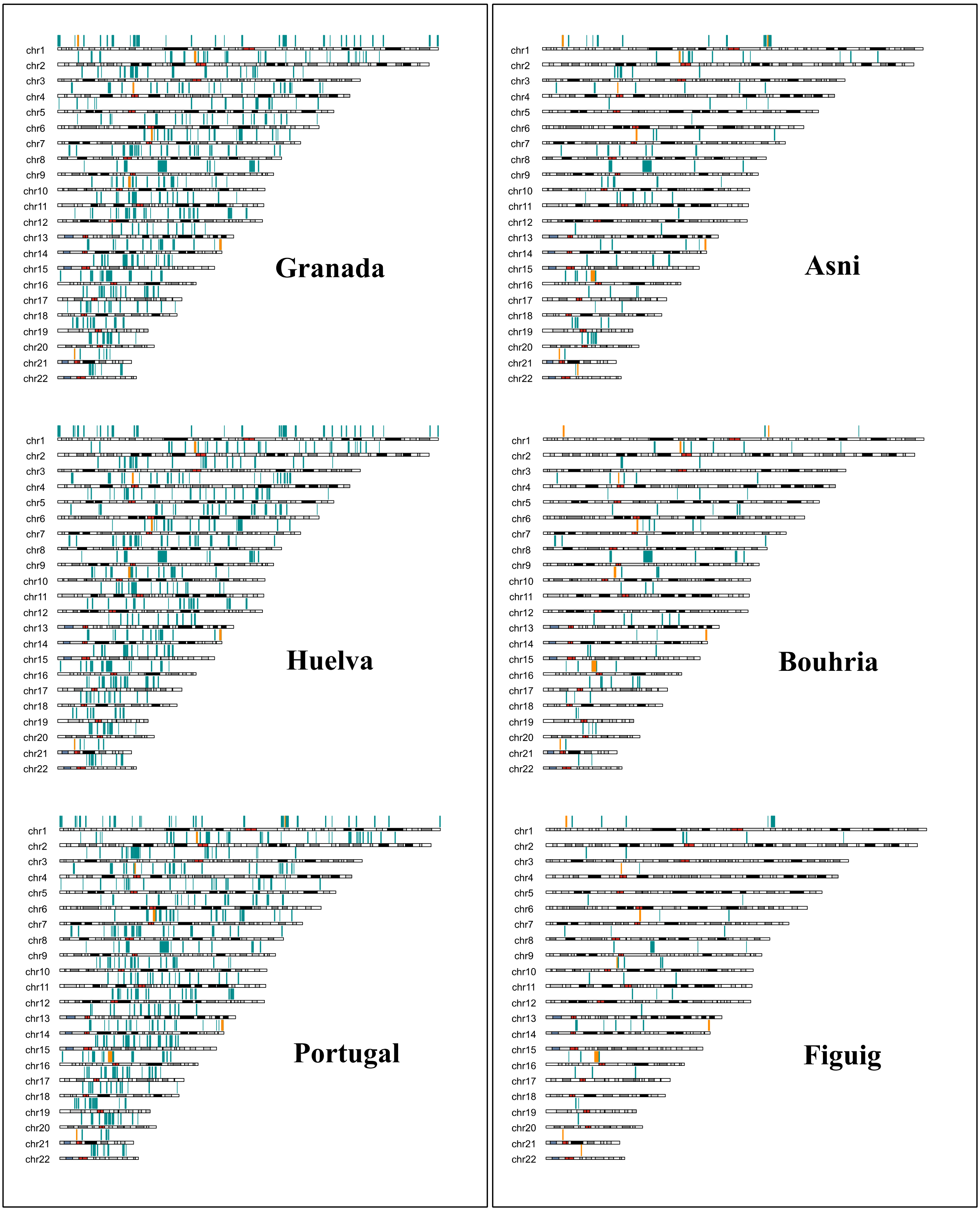


**Figure S1.** Karyograms showing ROHi genomic location in the western Mediterranean populations (*islands_step1*). Orange blocks represent those islands shared with at least other population from this sample set.

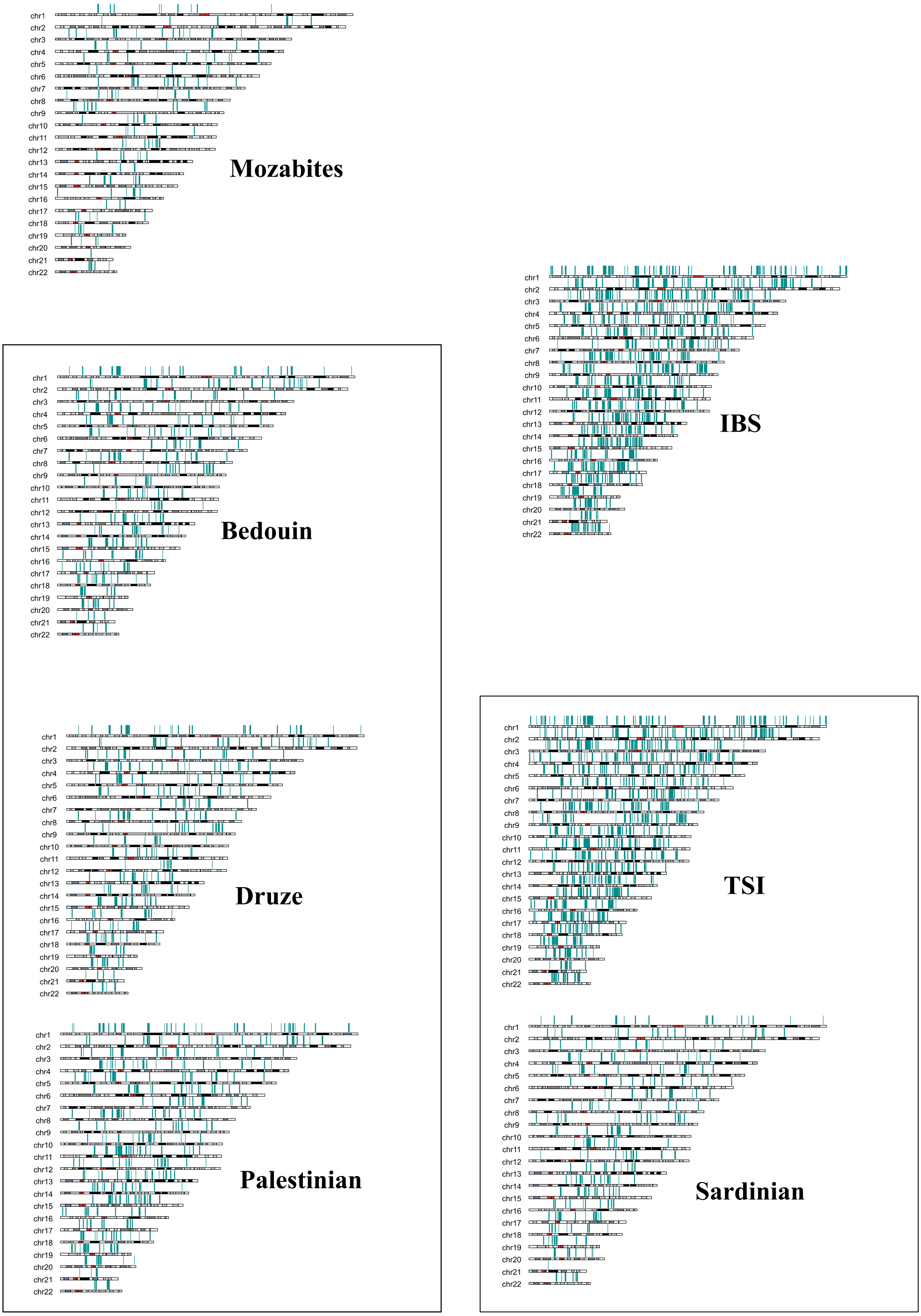


**Figure S2.** Karyograms showing ROHi genomic location in a selected set of Mediterranean populations taken from literature (*islands_step1*).

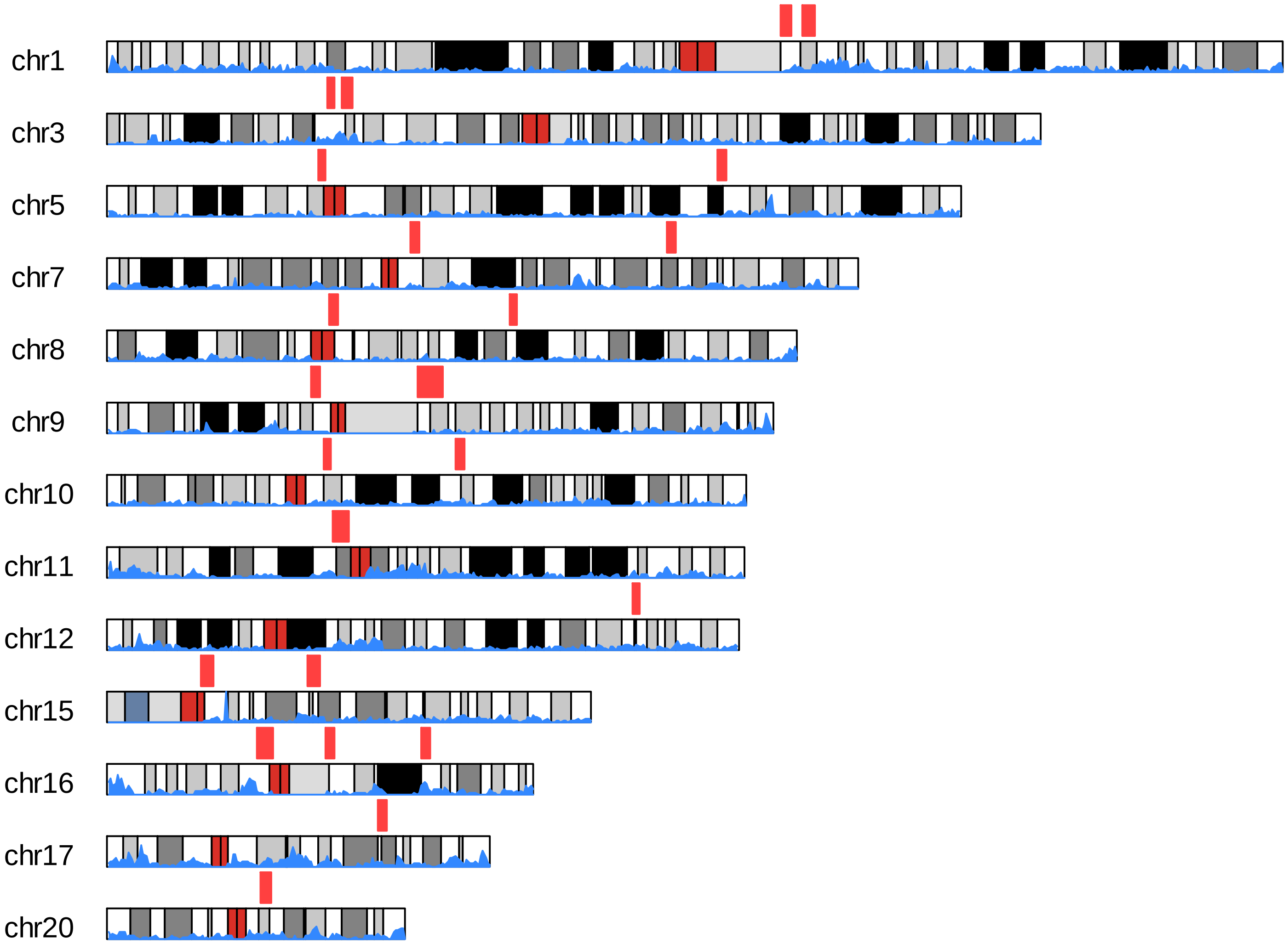


**Figure S3.** Genomic location of the biggest islands detected in *islands_step2* (metapopulation analysis) in the west Mediterranean. Gene density (in blue) is depicted within ideograms.

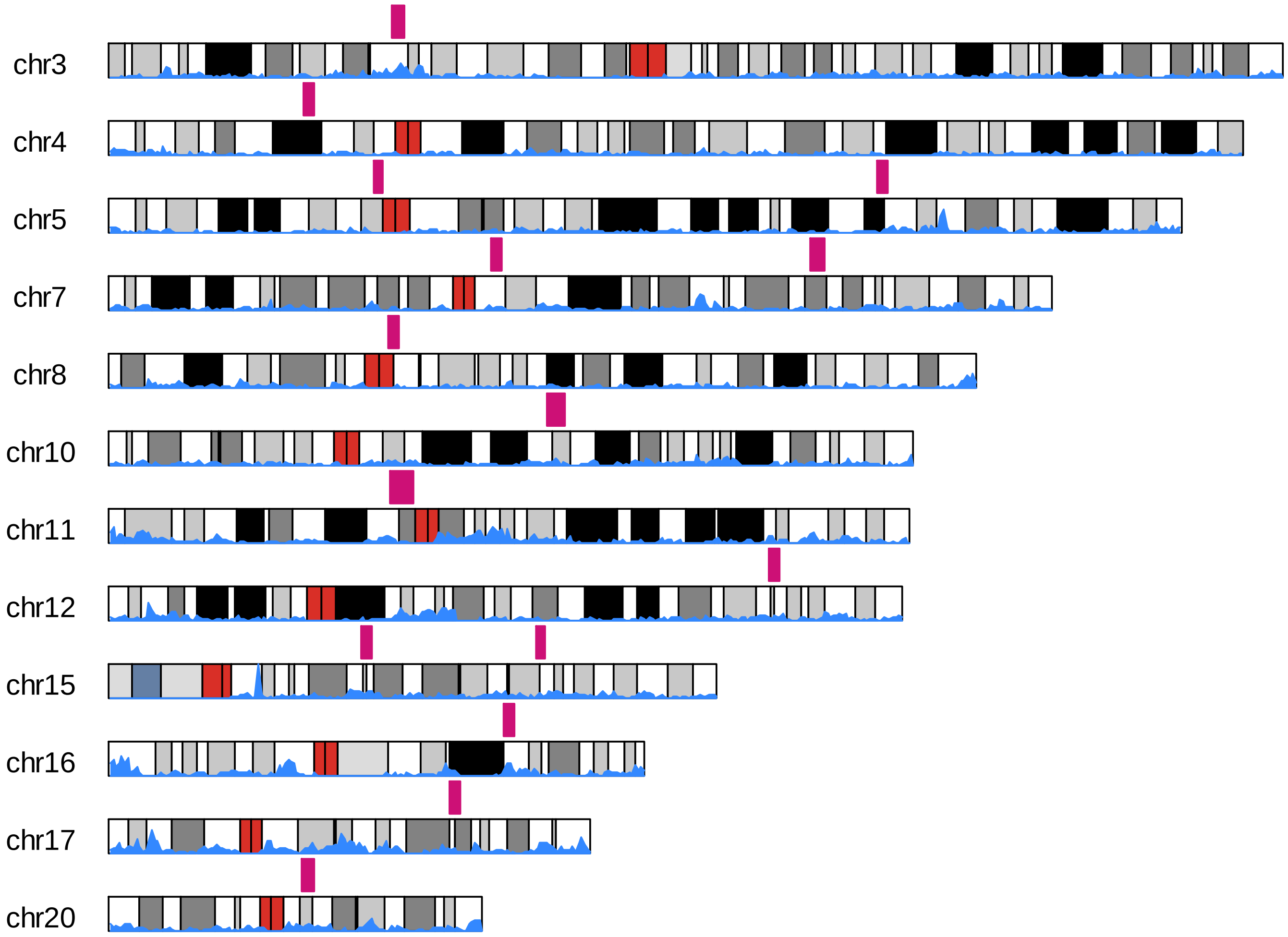


**Figure S4.** Genomic location of the biggest islands detected in *islands_step2* (metapopulation analysis) in the central Mediterranean. Gene density (in blue) is depicted within ideograms.

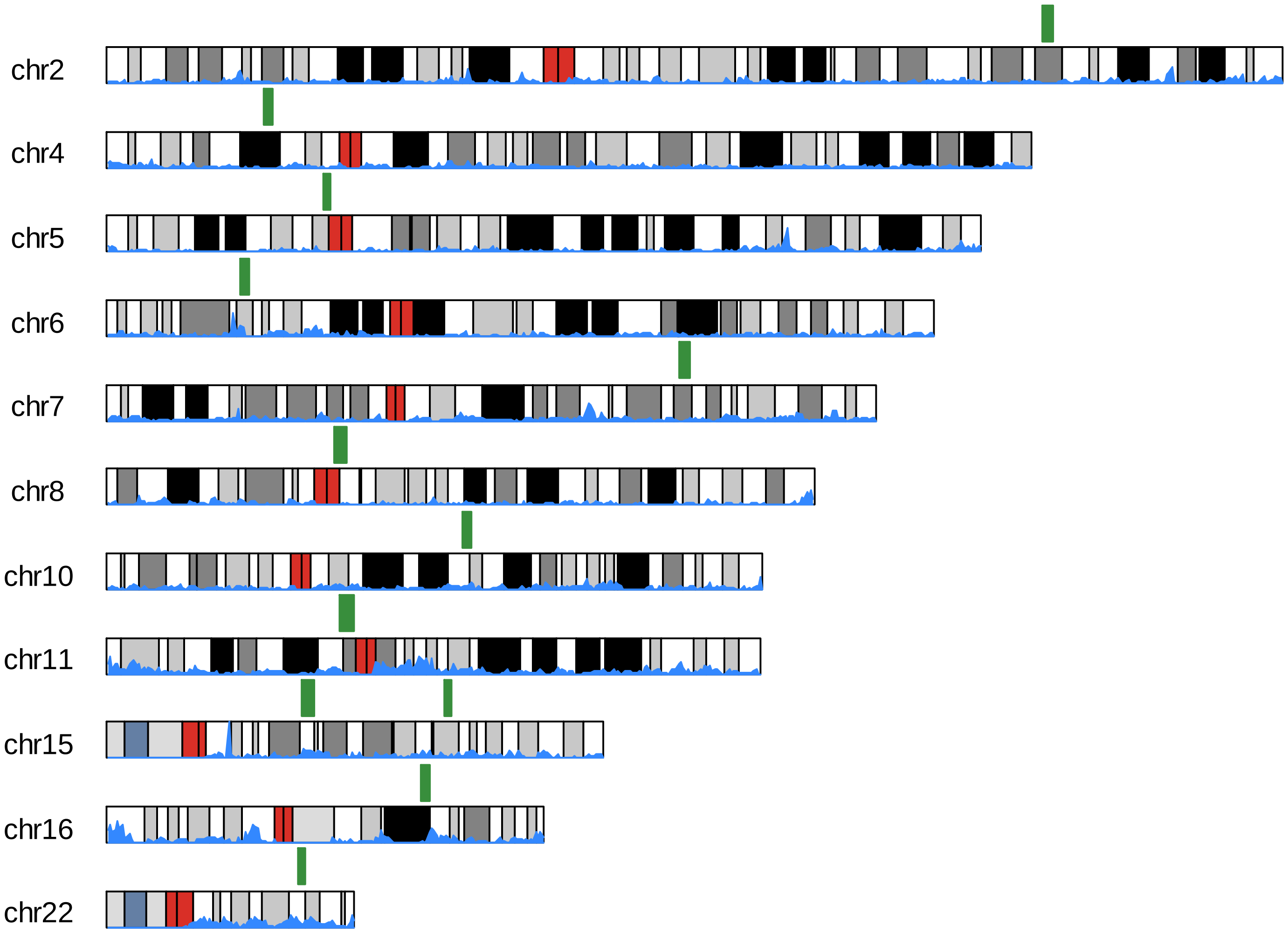


**Figure S5.** Genomic location of the biggest islands detected in *islands_step2* (metapopulation analysis) in the east Mediterranean. Gene density (in blue) is depicted within ideograms.
