# Supplementary material for: A genomic tale of inbreeding in western Mediterranean human populations
Source: Hum Genet. 2025 May 10;144(6):615–31. doi: 10.1007/s00439-025-02747-9 (PMC12401773; doi:10.1007/s00439-025-02747-9)
Supplement: Supplementary file 2 — Supplementary Material 2 [file 439_2025_2747_MOESM2_ESM.pdf]

Supplementary Tables

A genomic tale of inbreeding in western Mediterranean human populations

Candela L. Hernández, Luis J. Sánchez-Martínez, Francisco C. Ceballos, Jean-Michel Dugoujon, Luisa Pereira, Rosario Calderón

Table S1. Population database used for comparative purposes. 2

Table S2. Summary statistics of ROH fragments (populations from database) 3

Table S3. Mean and standard deviation for each ROH length category in western Mediterranean populations. 5

Table S4. Estimates of F_{ROH} against F_{IS}. 6

Table S5. ROH islands that record a high representation (>90%) within each population (*islands_step1*). 7

Table S6. ROH islands defined by their high length (>1.5 Mb) within each population (*islands_step1*). 11

Table S7. ROH islands defined by their high length (>1.5 Mb) within each metapopulation (*islands_step2*). 20

Table S8. ROH islands previously identified in the literature that have been detected in the present study 26

Table S1. Population database used for comparative purposes.

Acronym	Country	Population	Region	n	Genotype data	Reference
SPEA	*Spain*	*Eastern Andalusians*	South West Europe	35	Illumina Human Omni 2.5	Hernández et al. (2020)
SPWA	*Spain*	*Western Andalusians*	South West Europe	35	Illumina Human Omni 2.5	Hernández et al. (2020)
SPOR	*Portugal*	*Southern Portugal*	South West Europe	35	Illumina Human Omni 2.5	Hernández et al. (2020)
IBS	Spain	Spanish	South West Europe	99	Whole genome	1000 Genomes phase 3 release (Auton et al. 2015)
TSI	Italy	Tuscan	Central Mediterranean Europe	92	Whole genome	1000 Genomes phase 3 release (Auton et al. 2015)
SARD	Italy	Sardinian	Central Mediterranean Europe	28	Whole genome	HGDP-CEPH - new release / Bergström et al. (2020)
MAAS	*Morocco*	*Asni*	North Africa	15	Illumina Human Omni 2.5	Hernández et al. (2020)
MABO	*Morocco*	*Bouhria*	North Africa	10	Illumina Human Omni 2.5	Hernández et al. (2020)
MAFI	*Morocco*	*Figuig*	North Africa	9	Illumina Human Omni 2.5	Hernández et al. (2020)
DZMO	Algeria	Mozabite	North Africa	27	Whole genome	HGDP-CEPH - new release / Bergström et al. (2020)
ISBE	Israel	Bedouin (Negev)	Near East	46	Whole genome	HGDP-CEPH - new release / Bergström et al. (2020)
ISDR	Israel	Druze (Carmel)	Near East	42	Whole genome	HGDP-CEPH - new release / Bergström et al. (2020)
ISPA	Israel	Palestinian (Central)	Near East	46	Whole genome	HGDP-CEPH - new release / Bergström et al. (2020)

References

Auton, A. et al. (2015). A global reference for human genetic variation. *Nature*, 526(7571): 68–74.
Bergström, A. et al. (2020). Insights into human genetic variation and population history from 929 diverse genomes. *Science*, 367(6484): eaay5012.
Hernández, C. L. et al. (2020). Human genomic diversity where the Mediterranean joins the Atlantic. *Molecular Biology and Evolution*, 37(4): 1041–1055.

Table S2. Summary statistics on the structure of ROH fragments in a selected group of Mediterranean populations (see references in **Table S1**). Within the table, in bold, the total/mean values of each variable per population are shown.

	n	ROH Length Category (Mb)	Number of individuals (%)	Total ROH number (%)	Mean number of ROH per individual	Total ROH Length (Mb) per population (%)	Total ROH length (Mb) per individual	Mean ROH length (Mb)
**Iberian Peninsula**	99	ROH1 (0.3-0.5)	99/99 (100)	33323 (66.08)	336.59	12596.72 (48.09)	127.24	0.38
		ROH2 (0.5-1)	99/99 (100)	14549 (28.85)	146.96	9567.77 (36.52)	96.64	0.66
		ROH3 (1-2)	99/99 (100)	2271 (4.50)	22.94	2897.05 (11.06)	29.26	1.27
		ROH4 (2-4)	85/99 (85.86)	211 (0.42)	2.13	545.3 (2.08)	5.51	2.58
		ROH5 (4-8)	27/99 (27.27)	48 (0.10)	0.48	257.4 (0.98)	2.60	5.36
		ROH6 (>8)	13/99 (13.13)	26 (0.05)	0.26	331.36 (1.26)	3.35	12.74
				**50429**	**509.38**	**26195.91**	**264.60**	**0.52**
**Central Italy (Tuscany)**	92	ROH1 (0.3-0.5)	92/92 (100)	30980 (66.52)	336.74	11708.43 (49.45)	127.27	0.38
		ROH2 (0.5-1)	92/92 (100)	13491 (28.97)	146.64	8882.42 (37.52)	96.55	0.66
		ROH3 (1-2)	92/92 (100)	1930 (4.14)	20.98	2435.49 (10.29)	26.47	1.26
		ROH4 (2-4)	68/92 (73.91)	132 (0.28)	1.43	325.09 (1.37)	3.53	2.46
		ROH5 (4-8)	19/92 (20.65)	26 (0.06)	0.28	140.18 (0.59)	1.52	5.39
		ROH6 (>8)	10/92 (10.87)	12 (0.03)	0.13	184.9 (0.78)	2.01	15.41
				**46571**	**506.21**	**23676.51**	**257.35**	**0.51**
**Insular Italy (Sardinia)**	28	ROH1 (0.3-0.5)	28/28 (100)	10668 (56.34)	381	4050.97 (47.72)	144.68	0.38
		ROH2 (0.5-1)	28/28 (100)	5193 (27.42)	185.46	3447.01 (36.35)	123.11	0.66
		ROH3 (1-2)	28/28 (100)	935 (4.94)	33.39	1201.18 (12.67)	42.90	1.28
		ROH4 (2-4)	28/28 (100)	123 (0.65)	4.39	335.96 (3.54)	12.00	2.73
		ROH5 (4-8)	19/28 (67.85)	38 (0.20)	1.36	210.83 (2.22)	7.53	5.55
		ROH6 (>8)	11/28 (39.28)	17 (0.09)	0.61	236.32 (2.49)	8.44	13.90
				**18936**	**676.29**	**9483.37**	**338.69**	**0.50**
**Mozabite**	27	ROH1 (0.3-0.5)	27/27 (100)	6940 (56.21)	257.04	2630.64 (37.12)	97.43	0.38
		ROH2 (0.5-1)	27/27 (100)	3229 (26.15)	119.59	2157.58 (30.45)	79.91	0.67
		ROH3 (1-2)	27/27 (100)	630 (5.10)	23.33	823.22 (11.62)	30.49	1.31
		ROH4 (2-4)	27/27 (100)	189 (1.53)	7.00	525.3 (7.41)	19.45	2.78
		ROH5 (4-8)	23/27 (85.18)	79 (0.64)	2.93	425.81 (6.01)	15.77	5.39
		ROH6 (>8)	17/27 (62.96)	38 (0.31)	1.41	523.09 (7.38)	19.37	13.77

	n	ROH Length Category (Mb)	Number of individuals (%)	Total ROH number (%)	Mean number of ROH per individual	Total ROH Length (Mb) per population (%)	Total ROH length (Mb) per individual	Mean ROH length (Mb)
				**12347**	**457.30**	**7086.41**	**262.46**	**0.57**
**Bedouin**	46	ROH1 (0.3-0.5)	46/46 (100)	14014 (55.54)	304.65	5318.6 (32.64)	115.62	0.38
		ROH2 (0.5-1)	46/46 (100)	6735 (26.69)	146.41	4442.64 (27.27)	96.58	0.66
		ROH3 (1-2)	46/46 (100)	1294 (5.13)	28.13	1709.18 (10.49)	37.16	1.32
		ROH4 (2-4)	45/46 (97.82)	289 (1.15)	6.28	786.86 (4.83)	17.11	2.72
		ROH5 (4-8)	37/46 (80.43)	181 (0.72)	3.93	1033.25 (6.34)	22.46	5.71
		ROH6 (>8)	37/46 (80.43)	184 (0.73)	4.00	3002.06 (18.42)	65.26	16.32
				**25231**	**548.50**	**16294.25**	**354.22**	**0.65**
**Druze**	42	ROH1 (0.3-0.5)	42/42 (100)	13814 (56.53)	328.90	5237.91 (36.58)	124.71	0.38
		ROH2 (0.5-1)	42/42 (100)	6564 (26.86)	156.29	4324.12 (30.20)	102.96	0.66
		ROH3 (1-2)	42/42 (100)	1102 (4.51)	26.24	1403.08 (9.80)	33.41	1.27
		ROH4 (2-4)	42/42 (100)	197 (0.81)	4.69	542.57 (3.79)	12.92	2.75
		ROH5 (4-8)	32/42 (76.19)	104 (0.43)	2.48	566.26 (3.95)	13.48	5.44
		ROH6 (>8)	27/42 (64.28)	129 (0.53)	3.07	2243.62 (15.67)	53.42	17.39
				**24436**	**581.81**	**14319.33**	**340.94**	**0.59**
**Palestinian**	46	ROH1 (0.3-0.5)	46/46 (100)	14403 (58.28)	313.11	5462.53 (41.05)	118.75	0.38
		ROH2 (0.5-1)	46/46 (100)	6529 (26.42)	141.93	4293.86 (32.27)	93.34	0.66
		ROH3 (1-2)	46/46 (100)	1043 (4.22)	22.67	1330.33 (10.00)	28.92	1.28
		ROH4 (2-4)	43/46 (93.48)	119 (0.48)	2.59	316.31 (2.38)	6.88	2.66
		ROH5 (4-8)	30/46 (65.22)	73 (0.30)	1.59	426.16 (3.20)	9.26	5.84
		ROH6 (>8)	25/46 (54.35)	89 (0.36)	1.93	1476.85 (11.10)	32.11	16.59
				**24713**	**537.24**	**13307.54**	**289.29**	**0.54**

Table S3. Mean and standard deviation (sd) for each ROH length category in western Mediterranean population samples represented in **Figure 1A**.

**Population**	**Mean Total Size (Mb)**					
	**ROH1 (0.3-0.5 Mb)** mean ± sd	**ROH2 (0.5-1 Mb)** mean ± sd	**ROH3 (1-2 Mb)** mean ± sd	**ROH4 (2-4 Mb)** mean ± sd	**ROH5 (4-8 Mb)** mean ± sd	**ROH6 (>8 Mb)** mean ± sd
E. Andal. (Granada)	154.62 ± 10.99	116.90 ± 12.17	38.88 ± 7.85	12.43 ± 5.18	6.03 ± 5.11	3.19 ± 24.29
W. Andal. (Huelva)	154.63 ± 9.85	118.45 ± 9.39	39.27 ± 9.60	12.93 ± 5.97	9.02 ± 7.97	9.94 ± 48.76
Southern Portugal	151.49 ± 9.03	113.15 ± 10.37	36.06 ± 8.35	11.60 ± 4.61	4.68 ± 4.11	5.02 ± 44.94
Iberia (1KG)	127.24 ± 6.26	96.64 ± 9.35	29.26 ± 7.23	5.51 ± 3.90	2.60 ± 5.40	3.35 ± 10.29
Asni (Moroccan Berbers)	120.46 ± 10.20	94.56 ± 9.05	34.99 ± 9.70	16.69 ± 7.00	10.84 ± 10.82	43.05 ± 48.90
Bouhria (Moroccan Berbers)	118.62 ± 12.67	86.28 ± 11.06	31.52 ± 6.93	17.16 ± 9.97	13.31 ± 12.75	62.02 ± 134.95
Figuig (Moroccan Berbers)	116.83 ± 8.96	93.50 ± 17.38	35.86 ± 6.77	25.40 ± 9.67	22.77 ± 9.69	47.40 ± 70.45

Table S4. Estimates of F_{ROH} against F_{IS} inbreeding coefficients. The populations analyzed in the present study are highlighted in italics.

		F_{ROH}		F_{IS}	
**Population**	**n**	**Mean**	**SD**	**Mean**	**SD**
E. Andal. (Granada) (SP)	35	0.0140	0.0086	-0.0136	0.0072
W. Andal. (Huelva) (SP)	35	0.0209	0.0182	-0.0096	0.0083
South Portugal (PT)	35	0.0168	0.0159	-0.0140	0.0084
Iberia (IB)	99	0.0062	0.0051	-0.0005	0.0079
Toscana (IT)	92	0.0044	0.0031	-0.0035	0.0062
Sardinia (IT)	28	0.0150	0.0078	-0.0098	0.0107
Asni Berbers (MO)	15	0.0293	0.0208	-0.0166	0.0290
Bouhria Berbers (MO)	10	0.0417	0.0503	-0.0280	0.0445
Figuig Berbers (MO)	9	0.0397	0.0282	-0.0490	0.0333
Mozabite Berbers (MO)	27	0.0236	0.0147	-0.0005	0.0192
Bedouin (IS)	46	0.0438	0.0292	0.0343	0.0401
Druze (Negel) (IS)	42	0.0349	0.0324	0.0148	0.0376
Palestinian (IS)	46	0.0214	0.0250	0.0054	0.0305

Table S5. ROH islands that record a high representation (>90%) within each population (*islands_step1*). Islands are ordered by chromosome location. Genes are listed alphabetically within each fragment. GO (gene ontology) enrichment represents those biological processes potentially enriched from a given gene list. Results are queried with FDR *p* <0.05 and top fold enrichment >10.

Chromosome	Start	End	Length (Mb)	Nº individuals (% within the population)	Population	Genes	GO enrichment
1	12900000	13700000	0.8	93.30<br>100.00<br>100.00<br>91.43	Morocco (Asni)<br>Morocco (Bouhria)<br>Morocco (Figuig)<br>E. Andalusia	HNRNPC genes, PRAMEF genes	negative regulation of cell differentiation, negative regulation of apoptotic process, negative regulation of DNA-templated transcription
1	147400000	147700000	0.3	100.00<br>90.00<br>91.40	Morocco (Asni)<br>Morocco (Bouhria)<br>S. Portugal	ABHD17AP1, GPR89B, NBPF24, PDZK1P2, PFN1P4, RNA5SP58	-
1	147900000	149800000	1.9	100.00	Morocco (Figuig)	DRD5P2, FCGR1 genes, HIST2H genes, NBPF genes, NKAIN1P1, PFN1P genes, PPIAL4 genes, RNA5SP59	protein peptidyl-prolyl isomerization, protein folding
2	89200000	90200000	1	93.30<br>100.00<br>97.14<br>97.14<br>94.30	Morocco (Asni)<br>Morocco (Bouhria)<br>E. Andalusia<br>W. Andalusia<br>S. Portugal	IGKV genes, PGBD4P5	immunoglobulin production, adaptive immune response
3	98500000	98800000	0.3	92.86	Sardinia	DCBLD2, LINC00973, PDLIM1P4, ST3GAL6	-
4	49000000	49600000	0.6	90.00<br>91.43<br>91.40<br>100.00<br>100.00<br>100.00	Morocco (Bouhria)<br>E. Andalusia<br>S. Portugal<br>Morocco (Asni)<br>Morocco (Figuig)<br>W. Andalusia	ANKRD20A17P, CWH43, SNX18P genes, TPI1P4	-

Chromosome	Start	End	Length (Mb)	Nº individuals (% within the population)	Population	Genes	GO enrichment
7	61100000	62000000	0.9	100.00<br>100.00<br>100.00<br>100.00<br>100.00	Morocco (Asni)<br>Morocco (Bouhria)<br>Morocco (Figuig)<br>E. Andalusia<br>W. Andalusia	-	-
7	61100000	62200000	1.1	91.40	S. Portugal	-	-
9	43400000	44100000	0.7	90.00	Morocco (Bouhria)	ANKRD20A7P, ATP5A1P5, CNN2P4, CNTNAP3B, CYP4F60P, RN7SL343P, SNX18P5, SPATA31A6	-
9	44200000	45300000	1.1	93.30	Morocco (Asni)	ATP5A1P6, FAM27C, FAM27E4, RBPJP6	-
10	46200000	47000000	0.8	100.00	Morocco (Figuig)	AGAP4, BMS1P1, CTGLF8P, CTSLP5, DUSP8P3, FAM genes, FRMPD2P2, GLUD1P7, GPRIN2, PTPN20A, RHEBP1, RN7SL248P, RNA5SP genes, SYT15	-
10	46200000	47500000	1.3	100.00<br>91.43	Morocco (Bouhria)<br>W. Andalusia	AGAP genes, ANXA8L1, BMS1P genes, CTGLF8P, CTSLP genes, DUSP8P genes, FAM genes, FRMPD2P2, GLUD1P genes, GPRIN2, HNRNPA1P33,NPY4R, PTPN20A, RHEBP genes, RN7SL genes, RNA5SP genes, SYT15	-
10	46200000	47600000	1.4	91.43	E. Andalusia	AGAP genes, AHCYP1, ANXA8L1, BMS1P genes, CTGLF8P, CTSLP genes, DUSP8P genes, FAM genes, FRMPD2P2, GLUD1P genes, GPRIN2, HNRNPA1P33, NPY4R, PTPN20A, RHEBP genes, RN7SL genes, RNA5SP genes, SYT15	-
10	51100000	51400000	0.3	100.00	Morocco (Figuig)	AGAP8, FAM21D, FAM25D, PARG, RNA5SP316, SNORA74, TIMM23B	-
14	106000000	107100000	1.1	94.29<br>97.10	E. Andalusia<br>S. Portugal	ADAM6, ATP5G1P1, ELK2AP, ELK2BP, HOMER2P genes, IGH genes, KIAA0125, RNA5SP357, SLC20A1P2	antibody-dependent cellular cytotoxicity, complement activation, classical pathway, B cell receptor signaling pathway, antibacterial humoral response

Chromosome	Start	End	Length (Mb)	Nº individuals (% within the population)	Population	Genes	GO enrichment
14	106000000	107200000	1.2	91.43	W. Andalusia	ADAM6, ATP5G1P1, ELK2 genes, HOMER2P genes, IGH genes, KIAA0125, RNA5SP357, SLC20A1P genes	antibody-dependent cellular cytotoxicity, complement activation, classical pathway, B cell receptor signaling pathway, antibacterial humoral response
14	106100000	107100000	1	100.00	Morocco (Asni)	ADAM6, ELK2AP, HOMER2P genes, IGH genes, RNA5SP357, SLC20A1P2	complement activation, classical pathway, B cell receptor signaling pathway, antibacterial humoral response
				100.00	Morocco (Bouhria)		
				100.00	Morocco (Figuig)		
16	31700000	34500000	2.8	90.00	Morocco (Bouhria)	ABCD1P3, ACTR3BP3, ARHGAP23P1, BMS1P8, CCNYL3, CLUHP3, ENPP7P13, FAM108A genes, FGFR3P5, HERC2P genes, IGH genes, PABPC1P13, PCMTD1P2, RARRES2P genes, RBM22P13, SLC25A1P4, SLC6A10P, TP53TG3 genes, UBE2MP1, VN1R3, VN1R genes, VN1R68P, VN1R69P, ZNF genes	immunoglobulin mediated immune response
16	31900000	34200000	2.3	93.30	Morocco (Asni)	ABCD1P3, ACTR3BP3, ARHGAP23P1, BMS1P8, ENPP7P13, FAM108A genes, HERC2P genes, PABPC1P13, PCMTD1P2, SLC6A10P, TP53TG3 genes, ZNF267	immunoglobulin mediated immune response
16	31900000	34300000	2.4	100.00	Morocco (Figuig)	ABCD1P3, ACTR3BP3, ARHGAP23P1, BMS1P8, CCNYL3, ENPP7P13, FAM108A genes, HERC2P genes, IGH genes, PABPC1P13, PCMTD1P2, SLC6A10P, TP53TG3 genes, ZNF267	immunoglobulin mediated immune response
				97.10	S. Portugal		

Chromosome	Start	End	Length (Mb)	Nº individuals (% within the population)	Population	Genes	GO enrichment
21	10700000	11100000	0.4	100.00	Morocco (Asni)	BAGE2, CYCSP41, SLC25A15P4, TPTE	-
				100.00	Morocco (Bouhria)		
				100.00	Morocco (Figuig)		
				97.14	E. Andalusia		
				100.00	W. Andalusia		
				100.00	S. Portugal		
22	22700000	23200000	0.5	93.30	Morocco (Asni)	ASH2LP1, BCRP4, GGTLC2, IGL genes, MIR650, POM121L1P, PRAME, ZNF280A, ZNF280B	immunoglobulin production, adaptive immune response
				100.00	Morocco (Figuig)		

Note: for the populations not recorded in the Table, the maximum values of ROHi representation within the populations are as follows: IBS 58%, TSI 62%, DZMO 64%, ISBE 37%, ISDR 78%, ISPA 67% (see **Table S1** for acronym description).

Table S6. ROH islands defined by their high length (>1.5 Mb) within each population (*islands_step1*). Islands are ordered first by size and then by chromosomic coordinates. Genes are listed alphabetically within each fragment. GO (gene ontology) enrichment represents those biological processes potentially enriched from a given gene list. Results are queried with FDR *p* <0.05 and top fold enrichment >10.

**Chromosome**	**Start**	**End**	**Length (Mb)**	**Frequency (%) of occurrence within the population**	**Population**	**Genes**	**GO enrichment**
9	65700000	71000000	5.3	66.70	Morocco (Asni)	ANKRD20A genes, AQP7P genes, ATP5A1P10, BMS1P genes, CBWD genes, CDK2AP2P genes, CNN2P genes, CYP4F genes, FAM genes, FOXD4 genes, MEP1AP2, MIR1299, MIR4477A, MYO5BP genes, PGM5 genes, PTGER4P genes, RBPJP2, RN7SL787P, RNA5SP genes, SNORA70	-
				80.00	Morocco (Bouhria)		
				85.71	E. Andalusia		
				82.86	W. Andalusia		
				82.90	S. Portugal		
9	68300000	70600000	2.3	88.90	Morocco (Figuig)	ANKRD20A4, AQP7P2, BMS1P genes, CBWD genes, CDK2AP2P3, CNN2P3, CYP4F25P, FOXD4 genes, MYO5BP3, PGM5P1, PGM5P2, PTGER4P3, RNA5SP284, SNX18P9	-
16	31900000	35200000	3.3	82.86	E. Andalusia	ABCD1P3, ACTR3BP3, AGGF1P4, ARHGAP23P1, BMS1P8, CCNYL3, ENPP7P13, FAM genes, FGFR3P5, HERC2P genes, HMGN2P41, IGH genes, PABPC1P13, PCMTD1P2, PPP1R1AP2, RARRES2P genes, RNA5SP genes, SLC25A1P4, SLC6A10P, TP53TG3 genes, UBE2MP1, VN1R genes, ZNF267	immunoglobulin mediated immune response

Chromosome	Start	End	Length (Mb)	Frequency (%) of occurrence within the population	Population	Genes	GO enrichment
16	31700000	34900000	3.2	85.71	W. Andalusia	ABCD1P3, ACTR3BP3, AGGF1P4, ARHGAP23P1, BMS1P8, CCNYL3, CLUHP3, ENPP7P13, FAM genes, FGFR3P5, HERC2P genes, IGH genes, PABPC1P13, PCMTD1P2, RARRES2P genes, RBM22P13, SLC25A1P4, SLC6A10P, TP53TG3 genes, UBE2MP1, VN1R genes, ZNF267, ZNF720	immunoglobulin mediated immune response
16	31700000	34500000	2.8	90.00	Morocco (Bouhria)	ABCD1P3, ACTR3BP3, ARHGAP23P1, BMS1P8, CCNYL3, CLUHP3, ENPP7P13, FAM108A genes, FGFR3P5, HERC2P genes, IGH genes, PABPC1P13, PCMTD1P2, RARRES2P genes, RBM22P13, SLC25A1P4, SLC6A10P, TP53TG3 genes, UBE2MP1, VN1R3, VN1R genes, VN1R68P, VN1R69P, ZNF genes	immunoglobulin mediated immune response
16	31900000	34200000	2.3	93.30	Morocco (Asni)	ABCD1P3, ACTR3BP3, ARHGAP23P1, BMS1P8, ENPP7P13, FAM108A genes, HERC2P genes, PABPC1P13, PCMTD1P2, SLC6A10P, TP53TG3 genes, ZNF267	immunoglobulin mediated immune response
16	31900000	34300000	2.4	100.00	Morocco (Figuig)	ABCD1P3, ACTR3BP3, ARHGAP23P1, BMS1P8, CCNYL3, ENPP7P13, FAM108A genes, HERC2P genes, IGH genes, PABPC1P13, PCMTD1P2, SLC6A10P, TP53TG3 genes, ZNF267	immunoglobulin mediated immune response
				97.10	S. Portugal		

Chromosome	Start	End	Length (Mb)	Frequency (%) of occurrence within the population	Population	Genes	GO enrichment
11	47400000	50600000	3.2	31.42	TSI	AGBL2, C1QTNF4, CBX3P8, CELF1, FAM180B, FNBP4, FOLH1, KBTBD4, MIR genes, MTCH2, NDUFS3, NUP160, OR4A genes, PSMC3, PTPMT1, PTPRJ, RAPSN, RN7SL652P, RNA5SP340, SLC39A13, SPI1, TRIM genes, TYRL, UBTFL7	detection of chemical stimulus involved in sensory perception of smell
11	48200000	51300000	3.1	40.53	Palestinian	CBX3P8, FOLH1, OR4A genes, TRIM genes, TYRL, UBTFL7	detection of chemical stimulus involved in sensory perception of smell
11	48200000	49700000	1.5	58.81	Sardinia	FOLH1, OR4 genes, TRIM genes	detection of chemical stimulus involved in sensory perception of smell
1	147400000	149800000	2.4	82.86	W. Andalusia	ABHD17AP1, DRD5P2, FAM genes, FCGR1 genes, GPR89B, HIST2H genes, NBPF genes, NKAIN1P1, PDZK1P2, PFN1P genes, PPIAL4 genes, RNA5SP genes	protein peptidyl-prolyl isomerization, protein folding
1	147800000	149800000	2	77.14<br>88.60	E. Andalusia<br>S. Portugal	DRD5P2, FAM genes, FCGR1 genes, FCGR1C, HIST2H genes, NBPF genes, NKAIN1P1, PFN1P genes, PPIAL4 genes, RNA5SP59	protein peptidyl-prolyl isomerization, protein folding
1	147900000	149800000	1.9	100.00	Morocco (Figuig)	DRD5P2, FCGR1 genes, HIST2H genes, NBPF genes, NKAIN1P1, PFN1P genes, PPIAL4 genes, RNA5SP59	protein peptidyl-prolyl isomerization , protein folding
1	147900000	149600000	1.7	73.30	Morocco (Asni)	FAM72C, HIST2H3PS2, NBPF genes, PPIAL4 genes	protein peptidyl-prolyl isomerization

Chromosome	Start	End	Length (Mb)	Frequency (%) of occurrence within the population	Population	Genes	GO enrichment
20	32500000	34800000	2.3	38.56	IBS	ACSS2, AHCY, ASIP, CDC42P1, CEP250, CNBD2, COX7BP2, CPNE1, DYNLRB1, EDEM2, EIF2S2, EIF6, EPB41L1, ERGIC3, FAM83C, FDX1P1, FER1L4, GDF5 genes, GGT7, GSS, HIGD1AP16, HMGB3P genes, ITCH, MAP1LC3A, MIR genes, MMP24, MT1P3, MYH7B, NCOA6, NFS1, PHF20, PIGPP3, PIGU, PROCR, RALY, RBM genes, RN7SKP271, RNA5SP483, ROMO1, RPF2P1, RPL genes, RPS2P1, SCAND1, SNORD56, SPAG4, TP53INP2, TPM3P2, TRPC4AP, UQCC1, XPOTP1	-
3	47600000	49800000	2.2	32.51	TSI	AMIGO3, AMT, APEH, ARIH genes, ATRIP, BSN, CAMP, CCDC genes, CELSR3, COL7A1, CSPG5, DAG1, DALRD3, DHX30, FBXW12, GMPPB, GPX1, IMPDH2, IP6K genes, KLHDC8B, LAMB2, MAP4, MST1, NCKIPSD, NDUFAF3, NICN1, NME6, P4HTM, PFKFB4, PLXNB1, PRKAR2A, QARS, QRICH1, RHOA, RNF123, SHISA5, SLC genes, SMARCC1, SPINK8, TCTA, TMA7, TMEM89, TREX1, UCN2, UQCRC1, USP19, USP4, WDR6, ZNF589	-
5	129600000	131800000	2.2	37.42	IBS	ACSL6, CDC42SE2, CSF2, FNIP1, HINT1, IL3, LYRM7, P4HA2, PDLIM4, RAPGEF6, SLC22A4, SLC22A5	-
5	129600000	131300000	1.7	32.23	TSI	ACSL6, CDC42SE2, FNIP1, HINT1, LYRM7, RAPGEF6	-
5	129700000	131300000	1.6	51.43<br>51.43	E. Andalusia<br>W. Andalusia	ACSL6, CDC42SE2, FNIP1, HINT1, LYRM7, RAPGEF6	-
5	129800000	131300000	1.5	61.67	Sardinia	ACSL6, CDC42SE2, FNIP1, HINT1, LYRM7, RAPGEF6	-

Chromosome	Start	End	Length (Mb)	Frequency (%) of occurrence within the population	Population	Genes	GO enrichment
7	64400000	66400000	2	31.46<br>30.33	IBS<br>TSI	ASL, CRCP, GUSB, KCTD7, RABGEF1, TMEM248, TPST1, VKORC1L1, ZNF genes	-
8	47000000	49000000	2	41.82<br>40.33	IBS<br>TSI	CEBPD, MCM4, PRKDC, SPIDR, UBE2V2	positive regulation of double-strand break repair, double-strand break repair, DNA recombination
8	47100000	49000000	1.9	48.57	E. Andalusia	CEBPD, MCM4, PRKDC, SPIDR, UBE2V2	positive regulation of double-strand break repair, double-strand break repair, DNA recombination
15	40300000	42300000	2	41.74	Bedouin	ANKRD63, BAHD1, BMF, BUB1B, CASC5, CHAC1, CHP1, CHST14, DISP2, DLL4, DNAJC17, EHD4, EIF2AK4, EXD1, GCHFR, INO80, ITPKA, IVD, JMJD7, KNSTRN, LTK, MAPKBP1, MGA, NDUFAF1, NUSAP1, OIP5, PAK6, PHGR1, PLA2G4 genes, PLCB2, PPP1R14D, RAD51, RHOV, RMDN3, RPAP1, RPUSD2, RTF1, SPINT1, SPTBN5, SRP14, TYRO3, VPS18, ZFYVE19	-
15	40500000	42200000	1.7	53.92	Druze	ANKRD63, BAHD1, BUB1B, CASC5, CHAC1, CHP1, CHST14, DISP2, DLL4, DNAJC17, EHD4, EXD1, GCHFR, INO80, ITPKA, IVD, JMJD7, KNSTRN, LTK, MAPKBP1, MGA, NDUFAF1, NUSAP1, OIP5, PAK6, PHGR1, PLA2G4B, PLCB2, PPP1R14D, RAD51, RHOV, RMDN3, RPAP1, RPUSD2, RTF1, SPINT1, SPTBN5, TYRO3, VPS18, ZFYVE19	-
7	118700000	120600000	1.9	51.43	E. Andalusia	ING3, KCND2, TSPAN12	-
9	43400000	45300000	1.9	77.14<br>74.29<br>77.10	E. Andalusia<br>W. Andalusia<br>S. Portugal	CNTNAP3B, FAM27C, SPATA31A6	-

Chromosome	Start	End	Length (Mb)	Frequency (%) of occurrence within the population	Population	Genes	GO enrichment
16	66500000	68400000	1.9	33.70	TSI	ACD, AGRP, ATP6V0D1, B3GNT9, BEAN1, CA7, CBFB, CCDC79, CDH16, CENPT, CES genes, DPEP genes, DUS2, DYNC1LI2, E2F4, EDC4, ELMO3, ENKD1, ESRP2, EXOC3L1, FAM genes, FBXL8, FHOD1, GFOD2, HSD11B2, HSF4, KCTD19, KIAA0895L, LCAT, LRRC genes, NAE1, NFATC3, NOL3, NRN1L, NUTF2, PARD6A, PDP2, PLA2G15, PLEKHG4, PRMT7, PSKH1, PSMB10, RANBP10, RLTPR, RRAD, SLC genes, SMPD3, THAP11, TK2, TMEM208, TPPP3, TRADD, TSNAXIP1, ZDHHC1	-
16	66700000	68400000	1.7	34.05	IBS	ACD, AGRP, ATP6V0D1, B3GNT9, CA7, CBFB, CCDC79, CDH16, CENPT, CES genes, CMTM4, CTC-479C5.12, CTCF, CTRL, DDX28, DPEP genes, DUS2, DYNC1LI2, E2F4, EDC4, ELMO3, ENKD1, ESRP2, EXOC3L1, FAM genes, FBXL8, FHOD1, GFOD2, HSD11B2, HSF4, KCTD19, KIAA0895L, LCAT, LRRC genes, NAE1, NFATC3, NOL3, NRN1L, NUTF2, PARD6A, PDP2, PLA2G15, PLEKHG4, PRMT7, PSKH1, PSMB10, RANBP10, RLTPR, RRAD, SLC genes, SMPD3, THAP11, TMEM208, TPPP3, TRADD, TSNAXIP1, ZDHHC1	-
16	65300000	66900000	1.6	39.81	Mozabite	BEAN1, CA7, CCDC79, CDH5, CKLF, CMTM genes, DYNC1LI2, NAE1, TK2	-
16	65300000	66800000	1.5	42.06	Druze	BEAN1, CCDC79, CDH5, CKLF, CMTM genes, DYNC1LI2, TK2	-

Chromosome	Start	End	Length (Mb)	Frequency (%) of occurrence within the population	Population	Genes	GO enrichment
15	42600000	44400000	1.8	35.45	TSI	ADAL, CAPN3, CATSPER2, CCNDBP1, CDAN1, CKMT1 genes, ELL3, EPB42, FRMD5, GANC, HAUS2, HYPK, LCMT2, LRRC57, MAP1A, MFAP1, PDIA3, PPIP5K1, SERF2, SERINC4, SNAP23, STARD9, STRC, TGM genes, TMEM62, TP53BP1, TTBK2, TUBGCP4, UBR1, WDR76, ZNF106, ZSCAN29	-
15	42800000	44300000	1.5	34.34	IBS	ADAL, CATSPER2, CCNDBP1, CDAN1, CKMT1 genes, ELL3, EPB42, FRMD5, HAUS2, HYPK, LCMT2, LRRC57, MAP1A, MFAP1, PDIA3, PPIP5K1, SERF2, SERINC4, SNAP23, STARD9, STRC, TGM genes, TMEM62, TP53BP1, TTBK2, TUBGCP4, UBR1, WDR76, ZSCAN29	-
15	43000000	44500000	1.5	45.71	E. Andalusia	ADAL, CATSPER2, CCNDBP1, CDAN1, CKMT1 genes, ELL3, EPB42, FRMD5, HYPK, LCMT2, MAP1A, MFAP1, PDIA3, PPIP5K1, SERF2, SERINC4, STARD9, STRC, TGM genes, TMEM62, TP53BP1, TTBK2, TUBGCP4, UBR1, WDR76, ZSCAN29	-
8	85200000	86900000	1.7	30.60	IBS	CA genes, E2F5, LRRCC1, RALYL, REXO1L genes	one-carbon metabolic process
17	57600000	59300000	1.7	43.37<br>48.08	IBS<br>TSI	APPBP2, BCAS3, CA4, CLTC, DHX40, HEATR6, PPM1D, PTRH2, RNFT1, RPS6KB1, TUBD1, USP32, VMP1	-
5	44700000	46300000	1.6	51.34<br>31.44	Druze<br>IBS	HCN1, MRPS30	-

Chromosome	Start	End	Length (Mb)	Frequency (%) of occurrence within the population	Population	Genes	GO enrichment
				46.09	IBS		
10	74100000	75700000	1.6	60.00	S. Portugal	AGAP5, ANXA7, CAMK2G, CHCHD1, DNAJB12, DNAJC9, ECD, FAM149B1, FUT11, MCU, MICU1, MRPS16, MSS51, MYOZ1, NDST2, NUDT13, OIT3, P4HA1, PLA2G12B, PLAU, PPP3CB, SEC24C, SYNPO2L, TTC18, USP54, ZSWIM8	-
				50.14	TSI		
10	74200000	75700000	1.5	60.00	E. Andalusia	AGAP5, ANXA7, CAMK2G, CHCHD1, DNAJC9, ECD, FAM149B1, FUT11, MCU, MICU1, MRPS16, MSS51, MYOZ1, NDST2, NUDT13, OIT3, P4HA1, PLA2G12B, PLAU, PPP3CB, SEC24C, SYNPO2L, TTC18, USP54, ZSWIM8	
10	73700000	75200000	1.5	46.23	Palestinian	ANAPC16, ANXA7, ASCC1, CHST3, DDIT4, DNAJB12, DNAJC9, ECD, FAM149B1, MCU, MICU1, MRPS16, MSS51, NUDT13, OIT3, P4HA1, PLA2G12B, PPP3CB, SPOCK2, TTC18	-
				59.86	Bedouin		
4	32900000	34400000	1.5	57.30	Druze	-	-
				62.62	Sardinia		
10	46100000	47600000	1.5	88.60	S. Portugal	AGAP genes, ANXA8L1, FAM genes, GPRIN2, NPY4R, PTPN20A, SYT15, ZFAND4	
12	111500000	113000000	1.5	57.14	E. Andalusia	ACAD10, ALDH2, ATXN2, BRAP, CUX2, ERP29, FAM109A, HECTD4, MAPKAPK5, NAA25, PTPN11, RPL6, SH2B3, TMEM116, TRAFD1	-
				43.41	TSI		

Chromosome	Start	End	Length (Mb)	Frequency (%) of occurrence within the population	Population	Genes	GO enrichment
22	41100000	42600000	1.5	20.94	TSI	ACO2, CCDC134, CENPM, CHADL, CSDC2, CYP2D6, DESI1, DNAJB7, EP300, FAM109B, L3MBTL2, MEI1, NAGA, NDUFA6, NHP2L1, PHF5A, PMM1, POLR3H, RANGAP1, RBX1, SEPT3, SLC25A17, SHISA8, SMDT1, SREBF2, ST13, TCF20, TEF, TNFRSF13C, TOB2, WBP2NL, XPNPEP3, XRCC6, ZC3H7B	-

Table S7. ROH islands defined by their high length (>1.5 Mb) within each metapopulation (*islands_step2*). Islands are ordered first by size and then by chromosomic coordinates. Genes are listed alphabetically within each fragment. GO (gene ontology) enrichment represents those biological processes potentially enriched from a given gene list. Results are queried with FDR *p* <0.05 and top fold enrichment >10. Colors are shown mirroring those depicted in **Figure 6**.

Chromosome	Start	End	Length (Mb)	Frequency (%) of occurrence within the population	Population	Genes	GO enrichment
9	65700000	71000000	5.3	80.56	W. Med	ANKRD20A genes, AQP7P genes, ATP5A1P10, BMS1P genes, BMS1P9, CBWD genes, CDK2AP2P genes, CNN2P genes, CYP4F genes , CYP4F61P, FAM genes, FOXD4 genes, MEP1AP2, MIR1299, MIR4477A, MYO5BP genes, PGM5 genes, PTGER4P genes, RBPJP2, RN7SL787P, RNA5SP genes, SNORA70	-
16	31700000	35200000	3.5	80.22	W. Med	ABCD1P3, ACTR3BP3, AGGF1P4, ARHGAP23P1, BMS1P8, CCNYL3, CLUHP3, ENPP7P13, FAM genes, FGFR3P5, HERC2P genes, IGH genes, PABPC1P13, PCMTD1P2, RARRES2P genes, RBM22P13, SLC25A1P4, SLC6A10P, TP53TG3 genes, UBE2MP1, VN1R genes, ZNF267, ZNF720	immunoglobulin mediated immune response
11	47300000	51300000	4	33.23	C. Med	AGBL2, C1QTNF4, CELF1, FAM180B, FNBP4, FOLH1, KBTBD4, MADD, MTCH2, MYBPC3, NDUFS3, NUP160, OR4 genes, PSMC3, PTPMT1, PTPRJ, RAPSN, SLC39A13, SPI1, TRIM genes	detection of chemical stimulus involved in sensory perception of smell
11	48000000	51300000	3.3	37.41	W. Med	FOLH1, OR4 genes, PTPRJ, TRIM genes	detection of chemical stimulus involved in sensory perception of smell, G protein-coupled receptor signaling pathway
11	48200000	51300000	3.1	34.76	E. Med	FOLH1, OR4 genes, TRIM genes	detection of chemical stimulus involved in sensory perception of smell, G protein-coupled receptor signaling pathway

Chromosome	Start	End	Length (Mb)	Frequency (%) of occurrence within the population	Population	Genes	GO enrichment
10	73900000	76800000	2.9	40.55	C. Med	ADK, AGAP5, ANAPC16, ANXA7, AP3M1, ASCC1, C10orf55, CAMK2G, CHCHD1, DDIT4, DNAJ genes, DUPD1, ECD, FAM149B1, FUT11, KAT6B, MCU, MICU1, MRPS16, MSS51, MYOZ1, NDST2, NUDT13, OIT3, P4HA1, PLA2G12B, PLAU, PPP3CB, SEC24C, SYNPO2L, TTC18, USP54, VCL, ZSWIM8	-
10	73900000	75700000	1.8	52.31	W. Med	AGAP5, ANAPC16, ANXA7, ASCC1, C10orf55, CAMK2G, CHCHD1, DDIT4, DNAJ genes, ECD, FAM149B1, FUT11, MCU, MICU1, MRPS16, MSS51, MYOZ1, NDST2, NUDT13, OIT3, P4HA1, PLA2G12B, PLAU, PPP3CB, SEC24C, SYNPO2L, TTC18, USP54, ZSWIM8	-
10	73600000	75300000	1.7	46.58	E. Med	ANAPC16, ANXA7, ASCC1, CHST3, DDIT4, DNAJ genes, ECD, FAM149B1, MCU, MICU1, MRPS16, MSS51, NUDT13, OIT3, P4HA1, PLA2G12B, PPP3CB, PSAP, SPOCK2, TTC18, USP54	-
15	20100000	22700000	2.6	27.28	W. Med	DKFZP547L112, GOLGA6L6, OR4 genes, POTEB genes	-
1	147400000	149900000	2.5	76.90	W. Med	ABHD17AP1, BOLA1, DRD5P2, FAM genes, FCGR1 genes, GPR89B, HIST2H genes, NBPF genes, NKAIN1P1, PFN1P genes, PPIAL4 genes, SF3B4, SV2A	protein peptidyl-prolyl isomerization, protein folding
1	142600000	144900000	2.3	27.46	W. Med	FAM72D, NBPF genes, PDE4DIP, PPIAL4 genes	-
8	47100000	49600000	2.5	36.00	E. Med	CEBPD, MCM4, PRKDC, SPIDR, UBE2V2	positive regulation of double-strand break repair, double-strand break repair, DNA recombination
8	46900000	49000000	2.1	42.49	W. Med	CEBPD, MCM4, PRKDC, SPIDR, UBE2V2	positive regulation of double-strand break repair, double-strand break repair, DNA recombination
8	47000000	49000000	2	36.58	C. Med	CEBPD, MCM4, PRKDC, SPIDR, UBE2V2	positive regulation of double-strand break repair, double-strand break repair, DNA recombination

Chromosome	Start	End	Length (Mb)	Frequency (%) of occurrence within the population	Population	Genes	GO enrichment
15	40300000	42800000	2.5	39.40	E. Med	ANKRD63, BAHD1, BMF, BUB1B, C15orf genes, CAPN3, CASC5, CHAC1, CHP1, CHST14, DISP2, DLL4, DNAJC17, EHD4, EIF2AK4, EXD1, GANC, GCHFR, INO80, ITPKA, IVD, JMJD7, KNSTRN, LTK, MAPKBP1, MGA, NDUFAF1, NUSAP1, OIP5, PAK6, PHGR1, PLA2G4 genes, PLCB2, PPP1R14D, RAD51, RHOV, RMDN3, RPAP1, RPUSD2, RTF1, SNAP23, SPINT1, SPTBN5, SRP14, TMEM87A, TYRO3, VPS genes, ZFYVE19, ZNF106	phosphatidylglycerol acyl-chain remodeling , glycerophospholipid catabolic process
15	42500000	45000000	2.5	36.17	W. Med	ADAL, CAPN3, CASC4, CATSPER2, CCNDBP1, CDAN1, CKMT1 genes, CTDSPL2, EIF3J, ELL3, EPB42, FRMD5, GANC, HAUS2, HYPK, LCMT2, LRRC57, MAP1A, MFAP1, PATL2, PDIA3, PPIP5K1, SERF2, SERINC4, SNAP23, SPG11, STARD9, STRC, TGM5, TGM7, TMEM genes, TP53BP1, TTBK2, TUBGCP4, UBR1, VPS39, WDR76, ZNF106, ZSCAN29	-
15	42500000	44400000	1.9	32.15	C. Med	ADAL, CAPN3, CATSPER2, CCNDBP1, CDAN1, CKMT1 genes, ELL3, EPB42, FRMD5, GANC, HAUS2, HYPK, LCMT2, LRRC57, MAP1A, MFAP1, PDIA3, PPIP5K1, SERF2, SERINC4, SNAP23, STARD9, STRC, TGM5, TGM7, TMEM genes, TP53BP1, TTBK2, TUBGCP4, UBR1, VPS39, WDR76, ZNF106, ZSCAN29	-
7	118200000	120500000	2.3	49.97	E. Med	KCND2, TSPAN12	-
7	118400000	120700000	2.3	39.60	C. Med	CPED1, ING3, KCND2, TSPAN12	-
7	118600000	120700000	2.1	40.41	W. Med	CPED1, ING3, KCND2, TSPAN12	-
20	32500000	34800000	2.3	29.38	C. Med	ACSS2, AHCY, ASIP, CDC42P1, CEP250, CNBD2, COX7BP2, CPNE1, DYNLRB1, EDEM2, EIF2S2, EIF6, EPB41L1, ERGIC3, FAM83C, FDX1P1, FER1L4, GDF5 genes, GGT7, GSS, HIGD1AP16, HMGB3P genes, ITCH, MAP1LC3A, MIR genes, MMP24, MT1P3, MYH7B, NCOA6, NFS1, PHF20, PIGPP3, PIGU, PROCR, RALY, RBM genes, RN7SKP271, RNA5SP483, ROMO1, RPF2P1, RPL genes, RPS2P1, SCAND1, SNORD56, SPAG4, TP53INP2, TPM3P2, TRPC4AP, UQCC1, XPOTP1	-
20	32500000	34800000	2.3	33.80	W. Med	ACSS2, AHCY, ASIP, CDC42P1, CEP250, CNBD2, COX7BP2, CPNE1, DYNLRB1, EDEM2, EIF2S2, EIF6, EPB41L1, ERGIC3, FAM83C, FDX1P1, FER1L4, GDF5 genes, GGT7, GSS, HIGD1AP16, HMGB3P genes, ITCH, MAP1LC3A, MIR genes, MMP24, MT1P3, MYH7B, NCOA6, NFS1, PHF20, PIGPP3, PIGU, PROCR, RALY, RBM genes, RN7SKP271, RNA5SP483, ROMO1, RPF2P1, RPL genes, RPS2P1, SCAND1, SNORD56, SPAG4, TP53INP2, TPM3P2, TRPC4AP, UQCC1, XPOTP1	-

Chromosome	Start	End	Length (Mb)	Frequency (%) of occurrence within the population	Population	Genes	GO enrichment
3	47600000	49800000	2.2	31.82	C. Med	AMIGO3, AMT, APEH, ARIH2 genes, ATRIP, BSN, CAMP, CCDC genes, CELSR3, COL7A1, CSPG5, DAG1, DALRD3, DHX30, FBXW12, GMPPB, GPX1, IMPDH2, IP6K genes, KLHDC8B, LAMB2, MAP4, MST1, NCKIPSD, NDUFAF3, NICN1, NME6, P4HTM, PFKFB4, PLXNB1, PRKAR2A, QARS, QRICH1, RHOA, RNF123, SHISA5, SLC genes, SMARCC1, SPINK8, TCTA, TMA7, TMEM89, TREX1, UCN2, UQCRC1, USP19, USP4, WDR6, ZNF589	-
3	46800000	48300000	1.5	33.94	W. Med	CAMP, CCDC12, CDC25A, CSPG5, DHX30, ELP6, KIF9, KLHL18, MAP4, MYL3, NBEAL2, PRSS genes, PTH1R, PTPN23, SCAP, SETD2, SMARCC1, ZNF589	-
3	49900000	52100000	2.2	38.76	W. Med	ABHD14 genes, ACY1, C3orf18, CACNA2D2, CAMKV, CISH, CYB561D2, DOCK3, DUSP7, GNA genes, GPR62, GRM2, HEMK1, HYAL genes, IFRD2, IQCF genes, LSMEM2, MANF, MAPKAPK3, MON1A, MST1R, NAT6, NPRL2, PARP3, PCBP4, RAD54L2, RASSF1, RBM genes, RPL29, RRP9, SEMA3F, TEX264, TMEM115, TUSC2, VPRBP, ZMYND10	cellular response to UV-B, hyaluronan catabolic process
4	32300000	34500000	2.2	49.05	E. Med	-	
4	32700000	34700000	2	45.96	C. Med	-	
7	64300000	66400000	2.1	29.01	C. Med	ASL, CRCP, GUSB, KCTD7, RABGEF1, TMEM248, TPST1, VKORC1L1, ZNF genes	-
7	64400000	66400000	2	30.04	W. Med	ASL, CRCP, GUSB, KCTD7, RABGEF1, TMEM248, TPST1, VKORC1L1, ZNF genes	-
2	193500000	195500000	2	36.94	E. Med	-	-
6	27500000	29400000	1.9	35.31	E. Med	GPX genes, HIST1H genes, NKAPL, OR genes, PGBD1, SCAND3, TRIM27, ZKSCAN genes, ZNF genes, ZSCAN genes	detection of chemical stimulus involved in sensory perception of smell
9	43400000	45300000	1.9	74.80	W. Med	CNTNAP3B, FAM27C, SPATA31A6	-

Chromosome	Start	End	Length (Mb)	Frequency (%) of occurrence within the population	Population	Genes	GO enrichment
16	46500000	48400000	1.9	36.88	W. Med	ABCC genes, C16orf87, DNAJA2, GPT2, ITFG1, LONP2, MYLK3, NETO2, ORC6, PHKB, SHCBP1, SIAH1, VPS35	-
16	66500000	68400000	1.9	31.05	C. Med	ACD, AGRP, ATP6V0D1, B3GNT9, BEAN1, CA7, CBFB, CCDC79, CDH16, CENPT, CES genes, CKLF, CMTM genes, CTCF, CTRL, DDX28, DPEP genes, DUS2, DYNC1LI2, E2F4, EDC4, ELMO3, ENKD1, ESRP2, EXOC3L1, FAM genes, FBXL8, FHOD1, GFOD2, HSD11B2, HSF4, KCTD19, KIAA0895L, LCAT, LRRC genes, NAE1, NFATC3, NOL3, NRN1L, NUTF2, PARD6A, PDP2, PLA2G15, PLEKHG4, PRMT7, PSKH1, PSMB10, RANBP10, RLTPR, RP11-5A19.5, RRAD, SLC genes, SMPD3, THAP11, TK2, TMEM208, TPPP3, TRADD, TSNAXIP1, ZDHHC1	-
16	65100000	66900000	1.8	37.35	E. Med	BEAN1, CA7, CCDC79, CDH11, CDH5, CKLF, CMTM genes, DYNC1LI2, NAE1, TK2	-
16	66700000	68400000	1.7	38.61	W. Med	ACD, AGRP, ATP6V0D1, B3GNT9, CA7, CBFB, CCDC79, CDH16, CENPT, CES genes, CMTM4, CTCF, CTRL, DDX28, DPEP genes, DUS2, DYNC1LI2, E2F4, EDC4, ELMO3, ENKD1, ESRP2, EXOC3L1, FAM genes, FBXL8, FHOD1, GFOD2, HSD11B2, HSF4, KCTD19, KIAA0895L, LCAT, LRRC genes, NAE1, NFATC3, NOL3, NRN1L, NUTF2, PARD6A, PDP2, PLA2G15, PLEKHG4, PRMT7, PSKH1, PSMB10, RANBP10, RLTPR, RRAD, SLC genes, SLC9A5, SMPD3, THAP11, TMEM208, TPPP3, TRADD, TSNAXIP1, ZDHHC1	-
5	129500000	131300000	1.8	37.82	C. Med	ACSL6, CDC42SE2, CHSY3, FNIP1, HINT1, LYRM7, RAPGEF6	-
				40.18	W. Med		
5	44700000	46400000	1.7	33.18	W. Med	HCN1, MRPS30	-
5	44700000	46300000	1.6	33.18	C. Med	HCN1, MRPS30	-
				44.12	E. Med		
8	85200000	86900000	1.7	33.80	W. Med	CA genes, E2F5, LRRCC1, RALYL, REXO1L genes	one-carbon metabolic process

Chromosome	Start	End	Length (Mb)	Frequency (%) of occurrence within the population	Population	Genes	GO enrichment
17	57600000	59300000	1.7	42.21	C. Med	APPBP2, BCAS3, CA4, CLTC, DHX40, HEATR6, PPM1D, PTRH2, RNFT1, RPS6KB1, TUBD1, USP32, VMP1	-
				48.88	W. Med		
12	111400000	113000000	1.6	33.65	C. Med	ACAD10, ALDH2, ATXN2, BRAP, CUX2, ERP29, FAM109A, HECTD4, MAPKAPK5, NAA25, PTPN11, RPL6, SH2B3, TMEM116, TRAFD1	-
10	46100000	47600000	1.5	84.93	W. Med	AGAP genes, ANXA8L1, FAM genes, GPRIN2, NPY4R, PTPN20A, SYT15, ZFAND4	-
12	111500000	113000000	1.5	43.71	W. Med	ACAD10, ALDH2, ATXN2, BRAP, CUX2, ERP29, FAM109A, HECTD4, MAPKAPK5, NAA25, PTPN11, RPL6, SH2B3, TMEM116, TRAFD1	-
15	69900000	71400000	1.5	45.02	E. Med	LARP6, LRRC49, THAP10, THSD4, TLE3, UACA	-
15	72100000	73600000	1.5	33.00	C. Med	ADPGK, ARIH1, BBS4, CELF6, GOLGA6B, GRAMD2, HEXA, HIGD2B, MYO9A, NEO1, PARP6, PKM, SENP8, TMEM202	-
22	39500000	41000000	1.5	23.83	E. Med	ADSL, APOBEC3H, ATF4, CACNA1I, CBX7, ENTHD1, FAM83F, GRAP2, MGAT3, MIEF1, MKL1, PDGFB, RPL3, RPS19BP1, SGSM3, SYNGR1, TAB1, TNRC6B	-

Table S8. ROH islands previously identified in the literature that have been detected in the present study. Start and end coordinates are expressed in kb. Only the function of those genes with potential evolutionary significance are listed. Gene enrichment results (GO) are highlighted in blue and the enrichment-fold values are provided. MF: *molecular function*; BP: *biological process*. The column "Populations" records the mean frequency values of each ROHi within populations.

Chromosome	Start	End	Genes (enrichment analysis)	Publication	Populations
1	52500	52800		present study	Morocco (Asni): 86.7%; Morocco (Figuig): 77.8%; S. Portugal: 71.4%; W. Andalusia: 74.3%; E. Andalusia: 68.6%
1	52500	53100		Ceballos et al. 2019	Africa: 34%
2	135800	136500		present study	E. Andalusia: 54.3%
2	135900	136800	lactase	Ceballos et al. 2019	Africa: 36.2%
2	136000	136200		present study	Morocco (Asni): 53.3%
2	136000	136300		present study	S. Portugal: 45.7%; W. Andalusia: 62.9%
2	136400	136700	lactase	present study	S. Portugal: 42.9%
2	136545	136594	lactase	Nothnagel et al. 2010	European
3	50382	51836	metabolism of dopaminergic neurons	Nothnagel et al. 2010	European
3	50600	51700	metabolism of dopaminergic neurons	present study	S. Portugal: 45.7%
3	50700	50800		present study	Morocco (Asni): 53.3%
3	50700	51900	metabolism of dopaminergic neurons	Ceballos et al. 2019	Africa: 20.7%
3	74900	75200		present study	S. Portugal: 40%; W. Andalusia: 51.4%; E. Andalusia: 42.9%
3	75000	75400		Ceballos et al. 2019	Africa: 28%
4	33300	34600		present study	S. Portugal: 60%
4	33300	34700		present study	E. Andalusia: 60%
4	33305	34167		Nothnagel et al. 2010	European
4	33305	34259		Pemberton et al. 2012	global: 34.3%
4	33400	34700		present study	W. Andalusia: 65.7%
4	52700	53000	spermatogenesis	present study	Morocco (Bouhria): 70%
4	52800	53200	spermatogenesis	Ceballos et al. 2019	Africa: 22.6%

Chromosome	Start	End	Genes (enrichment analysis)	Publication	Populations
5	44604	45448		Pemberton et al. 2012	global: 35.5%
5	44700	44900		present study	S. Portugal: 40%; W. Andalusia: 40%
5	45000	45100		present study	S. Portugal: 40%
5	45100	46400		present study	W. Andalusia: 57.1%
5	129700	131300		present study	W. Andalusia: 51.4%; E. Andalusia: 51.4%
5	129846	131423	interleukin	Nothnagel et al. 2010	European
5	137100	137900	myotilin	Ceballos et al. 2019	Africa: 22.6%
5	137100	137400	myotilin	present study	W. Andalusia: 40%
7	64400	66500	GO: amidine-lyase activity (MF, enrichment-fold >100)	Ceballos et al. 2019	Africa: 24.6%
7	64600	64700		present study	W. Andalusia: 37.1%
7	64900	66500	GO: amidine-lyase activity (MF, enrichment-fold >100)	Ceballos et al. 2019	Africa: 33%
7	65000	66500	GO: amidine-lyase activity (MF, enrichment-fold >100)	Ceballos et al. 2019	Africa: 26.5%
7	65100	66000	GO: amidine-lyase activity (MF, enrichment-fold >100)	Ceballos et al. 2019	Africa: 32.1%
7	65100	65300		present study	Morocco (Bouhria): 70%
7	65100	65400		present study	S. Portugal: 48.6%; E. Andalusia: 40%
8	67500	68100		present study	E. Andalusia: 62.9%
8	67500	68200		present study	S. Portugal: 65.7%
8	67600	68400		Ceballos et al. 2019	Africa: 35.6%
8	67600	67900		present study	Morocco (Asni): 60%
8	67600	68200		present study	W. Andalusia: 71.4%
8	67700	68100		present study	Morocco (Bouhria): 80%
9	125357	125762	olfactory receptors; GO: detection of chemical stimulus involved in sensory perception of smell (BP, fold=28.60); GO protein-coupled receptor signaling pathway (BP, fold=11.42); GO: olfactory receptor activity (MF, fold=28.73); GO: G protein-coupled receptor activity (MF, fold=16.07)	Pemberton et al. 2012	global: 36.7%
9	125400	126000	olfactory receptors; GO: detection of chemical stimulus involved in sensory perception of smell (BP, fold=21.45); GO protein-coupled receptor signaling pathway (BP, fold=10.15); GO: olfactory receptor activity (MF, fold=21.55); GO: G protein-coupled receptor activity (MF, fold=14.29)	present study	S. Portugal: 45.7%
9	125500	126000	olfactory receptors	present study	W. Andalusia: 54.3%

Chromosome	Start	End	Genes (enrichment analysis)	Publication	Populations
9	125600	125800		present study	Morocco (Bouhria): 70%
9	125600	125900		present study	E. Andalusia: 51.4%
10	73578	74430		Pemberton et al. 2012	global: 36.2%
10	74100	75700	GO: uniplex complex - calcium channel complex in the mitochondrial inner membrane (CC, fold>100)	present study	S. Portugal: 60%
10	74200	75700	GO: uniplex complex - calcium channel complex in the mitochondrial inner membrane (CC, fold>100)	present study	E. Andalusia: 60%
10	74212	75087	GO: uniplex complex - calcium channel complex in the mitochondrial inner membrane (CC, fold>100)	Nothnagel et al. 2010	European
11	47998	49391	olfactory receptors; GO: detection of chemical stimulus involved in sensory perception of smell (BP, fold=29.58); GO protein-coupled receptor signaling pathway (BP, fold=10.5); GO: olfactory receptor activity (MF, fold=29.71); GO: G protein-coupled receptor activity (MF, fold=14.77)	Nothnagel et al. 2010	European
11	48700	49100		present study	Morocco (Asni): 60%
11	66800	67200		present study	S. Portugal: 57.1%
11	66800	67300		present study	E. Andalusia: 60%
11	66900	67500		Ceballos et al. 2019	Africa: 33.3%
11	66900	67100		present study	Morocco (Asni): 53.3%; Morocco (Bouhria): 80%
11	66900	67200		present study	W. Andalusia: 74.3%
12	60400	60900		Ceballos et al. 2019	Africa: 21.9%
12	60600	60700		present study	E. Andalusia: 37.1%
12	86800	87000		present study	S. Portugal: 37.1%; W. Andalusia: 48.6%
12	86938	87756		Pemberton et al. 2012	global: 34.4%
12	87400	87600		present study	W. Andalusia: 40%
12	110250	111462		Nothnagel et al. 2010	European
12	110700	110900		present study	S. Portugal: 40%; W. Andalusia: 40%
13	57700	58300	dopamine D4 receptors	Ceballos et al. 2019	Africa: 21.5%
13	57700	58000	dopamine D4 receptors	present study	Morocco (Bouhria): 50%
13	57900	58200		present study	S. Portugal: 31.4%
14	65755	66957		Nothnagel et al. 2010	European

Chromosome	Start	End	Genes (enrichment analysis)	Publication	Populations
14	65900	66100		present study	W. Andalusia: 34.3%
14	66000	66100		present study	E. Andalusia: 34.3%
14	66600	67100		present study	W. Andalusia: 45.7%
14	66600	67600		present study	E. Andalusia: 40%
14	66800	67900		Ceballos et al. 2019	Africa: 22.2%
15	44400	45000		Ceballos et al. 2019	Africa
15	44600	44700		present study	W. Andalusia: 42.9%
15	44600	45000		present study	E. Andalusia: 40%; S. Portugal: 42.9%
15	69500	69900		present study	W. Andalusia: 60%; E. Andalusia: 48.6%
15	69700	69900		present study	S. Portugal: 42.9%
15	69881	70571		Pemberton et al. 2012	global: 34.7%
16	14600	15600		Ceballos et al. 2019	Africa: 26.6%
16	14700	15100		present study	Morocco (Asni): 80%; S. Portugal: 62.9%; E. Andalusia: 48.6%
16	14800	14900		present study	Morocco (Bouhria): 80%; Morocco (Figuig): 66.7%
16	14800	15100		present study	W. Andalusia: 45.7%
16	18800	18900		present study	S. Portugal: 37.1%; W. Andalusia: 40%
16	18300	19000		Ceballos et al. 2019	Africa: 28.3%
17	45400	45900		Ceballos et al. 2019	Africa: 22.1%
17	45400	45800		present study	S. Portugal: 51.4%
17	45500	45700		present study	W. Andalusia: 40%
20	33546	34281		Pemberton et al. 2012	global: 37.2%
20	33900	34800		present study	W. Andalusia: 48.6%
20	34100	34800		present study	S. Portugal: 65.7%; E. Andalusia: 42.9%

References: Ceballos et al. (2019) *Hum Genet*, 138:1123–1142; Nothnagel et al. (2010) *Hum Mol Genet*, 19:2927–2935; Pemberton et al. (2012) *Am J Hum Genet*, 91:275–92.
